# White Box Implementations Using Non-Commutative Cryptography

**DOI:** 10.3390/s19051122

**Published:** 2019-03-05

**Authors:** Leandro Marin

**Affiliations:** Area of Applied Mathematics (DITEC), Faculty of Computer Science, University of Murcia, 30071 Murcia, Spain; leandro@um.es; Tel.: +34-868887967

**Keywords:** white box cryptology, group-based cryptology, non-commutative cryptology, code obfuscation, IoT Security, fog computation, cloud computation

## Abstract

In this paper, we present a method to create a safe arithmetic that can be used to obfuscate implementations that require operations over commutative groups. The method is based on the structure of the endomorphisms of certain extensions of the original commutative group. The endomorphisms of a commutative group are non-commutative (in general), thus we can use a non-commutative group to emulate the arithmetic of a commutative one. The techniques presented in this paper are very flexible and the programmer has a wide variety of options to obfuscate the algorithms. The system can be parameterized using conjugations, thus it is possible to generate a different arithmetic for each instance of the program with a change in the security parameters, even in cases in which this number is huge (for example, in IoT applications). The security of this method is based not only on the difficulty of the conjugacy search problem (in a harder version because only partial information about the groups is known by the attacker), but also in a number of extra options that can be chosen by the programmer. The paper explains the general method, analyzes its algebraic properties and provides detailed examples based on the vector spaces over $F_{2}$ and XOR operators.

## 1. Introduction

The most traditional framework for cryptography is a pair of users, Alice and Bob, where Alice wants to send a message *m* to Bob through a communication channel. Alice and Bob live in secure bubbles, but the outside world is insecure and other users can be listening to the channel, therefore they agree to two functions *E* and *D* such that D(E(m))=m and instead of sending *m*, Alice sends E(m). Bob knows the function *D* and can compute m=D(E(m)) but any listener through the insecure channel would get E(m) and this information is useless without the function *D*. The functions *E* and *D* can be parameterized by a value *k* (the key) such that the algorithms can be made public and, if the value *k* is kept safe, the communication is secure.

This framework (the channel is insecure but the functions *E* and *D* are computed in a safe environment) is not valid in many real life situations. Consider the following examples:

- A sensor network with multiple nodes in which an attacker could have full access to some of  them
- A virus or an intruder getting valuable information from a running system
- Cloud or fog computations in which the environment is not completely safe due to malicious nodes or vulnerabilities such as Meltdown, Spectre, etc.
- Content providers broadcasting encrypted data to users that could try to obtain decryption keys to illegally distribute them to other users
- Computer games with limited licenses that could be manipulated by the users to obtain upgraded versions for free

As we can see in these examples, the sensitive information can be the data used in the computation or even the algorithm itself. White box cryptography assumes that the attacker has full access to the hardware and software, and even in that insecure environment, the obfuscated software would not provide sensible information to the attacker.

To define clearly which information is available to the attacker and which design parameters are not visible, we consider that an obfuscated implementation has public and private elements. The code itself is one of these public elements. A system is considered safe if the private elements cannot be (easily) recovered using the public information. Although all the techniques explained in this paper can also be used to hide the algorithm, we consider the worst case in which the attacker knows the algorithm that is implemented.

This case is usually considered in white box cryptography, for example safe implementations of the Data Encryption Standard (DES) and the Advanced Encryption Standard (AES) have been a major target in the development of white box cryptography.

One of the first proposals for DES, made by Chow et al. in [1], attracted great interest, although it was almost immediately broken by Jacob et al. in [2] and Goubin et al. in [3]. Chow et al. also proposed a white-box implementation for AES in [4], broken by Billet et al. in [5]. The algorithms for DES and AES share a similar structure, they have several rounds with combinations of affine transformations, XOR operators and S-boxes. A general attack over this kind of algorithms applied to cases different from DES and AES was given by Michiels et al. in [6]. Another attack based on this structure was given by Biryukov et al. in [7].

One standard technique used in white box cryptography when we have an addition is to spread the information into pieces (called shares) that are combined using the addition and some hidden linear transformations. For example, suppose that a vector *v* is represented by two vector $v_{1}$ and $v_{2}$, and we have secret linear transformations $e_{1}$ and $e_{2}$ such that $v=e_{1}\left(v_{1}\right)+e_{2}\left(v_{2}\right)$. The attacker can see $v_{1}$ and $v_{2}$ but $e_{1}$ and $e_{2}$ remain secret. If $w=e_{1}\left(w_{1}\right)+e_{2}\left(w_{2}\right)$ is the representation of another vector, we know that $v+w=e_{1}\left(v_{1}+w_{1}\right)+e_{2}\left(v_{2}+w_{2}\right)$, thus the program can compute additions without revealing $e_{1}$ and $e_{2}$. The problem is that the combination of these techniques with nonlinear transformations (S-boxes) can be analyzed to recover information.

A milestone in the history of white box cryptography was the WhibOx challenges that took place in 2017 (see [8]). In this contest, the developers created white box implementations of the advanced encryption standard (AES) following certain rules. The result was that the 94 challenges presented were broken 877 times. All of them were broken, in many cases using known techniques including the ones published in the previously mentioned examples. These kinds of breaks were done in a short period of time. Only 21 required more than one day to be broken for the first time. In general, those were the ones that required more special and unpublished attacks.

The second most resistant implementation on that contest is based on some of the ideas presented in this paper, although this paper is not about that implementation because the method presented in this paper is more sophisticated and it is not limited to the conditions of that contest. More details about the challenge and the attack made to the first implementation can be seen in [9,10].

It is possible to consider several approaches to white-box implementations. One of them is to consider an algorithm as a boolean circuit and implement methods for the basic boolean operators. This  is the idea presented in [10]. In this paper, we assume that the algorithm requires an obfuscated arithmetic over a commutative group *V*. Vector spaces are commutative groups and they are our main examples, but the theoretical construction is done in general. The role played by square matrices in the case of vector spaces, is played by the endomorphisms e:V→V in the case of commutative groups.

To represent the elements, we combine endomorphisms and values in *V*, but instead of having invisible operators $e_{i}$ and visible values $v_{i}$, we have visible information related with the operators $e_{i}$ and invisible values $v_{i}$. There are many advantages in this new approach. First, we can have a huge number of endomorphisms even in small cases, for example a vector space with $2^{8}$ vectors has $2^{8^{2}}=2^{64}$ matrices and we only need a small number of them to represent all the vectors, thus we have a lot of freedom to choose the ones to be used. The actual matrices need not be revealed, only how they operate, and many subsets can have identical operation tables. Moving the representation from *V* to the side operators is a kind of nonlinear transformation similar to a logarithm. This non-linearity avoids the standard attacks based on linearity and new layers of security can be applied without risk. The problem is that we cannot apply the linearity to compute the addition, thus we have to learn how to add using this new representation. This can be done by introducing new operators that are different from traditional ones. This new arithmetic is also an advantage because it can be parameterized by choosing different subsets of transformations (something similar to having a free choice for the base of the logarithms). The new operators also require smaller tables than the standard ones because the growth of the tables is linear and not quadratic. Finally, the use of non-commutative subgroups given by endomorphisms forces the attacker to solve the conjugacy search problem (CSP), which is considered hard even for quantum computers; thus, systems based on non-commutative groups can be considered as post-quantum cryptosystems. Several protocols and cryptosystems have been developed in recent years based on non-commutative groups (see, for example, [11]).

This paper is structured as follows. After this Introduction, the theory is explained in two steps. The main public and private elements of the theory are explained in Section 2. The second step is given in Section 3, in which we show some mathematical properties of the theory that can be used for an attacker to analyze the system and protections against them. The method is discussed in Section 4. In that section, we include information about use cases and further applications, especially to IoT. In Section 5, we show a big example with the most advanced features. The conclusions are in Section 6. Section 7 has information about patents related with this research. Finally, the paper has two appendices. Appendix A gives some basic mathematical terminology that is used in the paper. Appendix B contains a small obfuscated implementation using the techniques explained in the paper, written in full detail.

## 2. Private Arithmetic (Basic Version)

In this section, we describe the mathematical structure of an obfuscated implementation using a private arithmetic. Cryptography and cryptanalysis are in many cases “cat-and-mouse” games, in which the cryptographer tries to mislead the attacker by breaking the original rules and the attacker also tries methods that were not originally considered by the cryptographer in order to analyze the system and reveal the secret information.

The intention of this section is to give the definitions that explain the structure of the proposed obfuscation in a logical way. However, these definitions and procedures can be made more flexible to include some traps or difficulties to the attacker. Examples of these traps could be that not all the elements of a group are used in actual computations and we can include some misbehavior in these elements. In this example, we cannot say that *A* is a commutative group if there are two elements in *A* that do not have a correct multiplication. These special cases are a nightmare for explanations and we have decided to start with a basic version without *tricks* and we consider them in Section 3.

The objective of this theory is to obfuscate algorithms or data used in mathematical computations. The computations take place in a commutative group *V* that is called the *external commutative group*. This group is considered public.

The idea behind the technique that we explain is to use another commutative group *M* that is called the *internal commutative group* to make the internal computations. This group is private and, in fact, is not visible in the implementation. The operations in *M* are done using tables that do not provide the usual group addition, but certain information that is used to compute other non-standard operators.

In this section, the relation between *M* and *V* is simple; we have a projection p:M→V. This is a surjective group homomorphism and every element v∈V is represented using values m∈M such that p(m)=v. This relation is much more complex in the non-basic version given in Section 3.

The tables and methods that let us compute with the elements of *M* are called *internal arithmetic*. We call *external arithmetic* any computation that requires the actual value in the external group *V* and not only the value over *M*. A private arithmetic is a combination of an internal and an external  arithmetic.

A *critical point* in the program is a place at which the actual value in *V* has to be used for the computation. For example, the input and the output of the program are critical points. The  computations that take place in between are called connected components. A more detailed explanation about the connected components is provided in Section 2.10.

We decompose the analysis in two parts: first we analyze the internal arithmetic. This arithmetic can be used for several implementations. The elements of the internal arithmetic are:

- Public:
  - Public Group (*G*)
  - Heading Spaces (*X*)
  - *G*-structure of the heading spaces (G×X→X)
  - Reduction Maps (r:X→Y)
  - Dissolving Maps ($D:X\to G^{n}$)
- Private:
  - Internal Commutative Group (*M*)
  - Correspondence between heading spaces and valid elements ($\pi_{X}$)
  - Extended Arithmetical Group ($H{⋊}_{\varphi }G$)
  - Action of $H{⋊}_{\varphi }G$ on *M*.
  - Base points (*B*)

The second part is the elements that depend on the implementation and the group *V* (the external arithmetic). Its elements are:

- Public:
  - The external commutative group *V*
  - The program
  - Input Tables
  - Output Table
  - Tables for non-linear operators
- Private:
  - Control values and deals
  - Extra affine transformations

We have already mentioned the external commutative group *V* and the internal commutative group *M*; the rest of the elements are analyzed in different subsections.

### 2.1. Public Group *G* (Public)

The arithmetic is ruled by a group *G*. This group is given up to isomorphism. Therefore, it is possible to give it with its multiplication table or with any other representation that let us compute with the elements of the group. The group *G* is in general non-commutative.

### 2.2. Extended Group $H{⋊}_{\varphi }G$ (Private)

We select a second group *H* and a group homomorphism φ:G→Aut(H) to build the semi-direct product of the groups, $H{⋊}_{\varphi }G$. The group *G* is public, but the extension of the group to $H{⋊}_{\varphi }G$ is not.

### 2.3. Representation of $H{⋊}_{\varphi }G$ Using Endomorphisms of *M* (Private)

One of the most critical points in the security of the obfuscation is how the values of $H{⋊}_{\varphi }G$ operate over the elements of *M*. This requires a group homomorphism $\psi :H{⋊}_{\varphi }G\to \mathrm{Aut}\left(M\right)$. This is known in the literature as a representation of the group $H{⋊}_{\varphi }G$ on *M*, but, in this paper, we use also the word representation for other constructions, for example the representation of an element of *V* using elements of *M*, or the representation of elements in *M* with other arithmetical expressions; therefore, we use the more generic term action of $H{⋊}_{\varphi }G$ on *M*, which is used when *M* has no additional structure, but is valid in general.

The group *G* is a subgroup of $H{⋊}_{\varphi }G$, thus the action ψ also induces an action of *G* on *M*.

Given two actions $\psi ,\psi^{\prime }:H{⋊}_{\varphi }G\to \mathrm{Aut}\left(M\right)$, these are equivalent if there exists a group automorphism t:M→M such that $\psi^{\prime }\left(u\right)=t^{-1}\psi \left(u\right)t$ for all $u\in H{⋊}_{\varphi }G$.

Equivalent actions generate different arithmetics using the same groups. Their similar structures make them difficult to recognize for an attacker.

We next present an example of these kinds of constructions. Let $M=F_{2}^{4}$ and $h=\left[\begin{array}{cccc} 1 & 1 & 0 & 0\\ 1 & 0 & 0 & 0\\ 0 & 0 & 1 & 1\\ 0 & 0 & 1 & 0 \end{array}\right]$ and $g=\left[\begin{array}{cccc} 0 & 1 & 0 & 0\\ 1 & 0 & 0 & 0\\ 0 & 0 & 0 & 1\\ 0 & 0 & 1 & 0 \end{array}\right]$.

Let *G* be the group generated by the matrix *g* and *H* the group generated by *h*. In this case, *G* has 2 elements and *H* has 3. The group *H* has a nontrivial automorphism τ:H→H given by $\tau \left(h\right)=h^{-1}$ and one possible homomorphism φ:G→Aut(H) would be $\varphi \left(g^{i}\right)=\tau^{i}$. The semi-direct product $H{⋊}_{\varphi }G$ is a group of six elements and $\psi :H{⋊}_{\varphi }G\to \mathrm{Aut}\left(M\right)$ is given using the matrices *g* and *h* that we have defined previously.

The group Aut(M) is the group of invertible matrices 4×4 over $F_{2}$. This group has 20,160 elements and, using invertible matrices *t* to generate other actions equivalent to ψ, we get 540 essentially different actions of $H{⋊}_{\varphi }G$ on *M*.

### 2.4. Heading Spaces (Public)

A heading space is a set *X* with an action G×X→X. It is not even necessary that the set *X* has an additive structure; it can be any set that could be operated with the elements of *G*. We can have one or more heading spaces.

In this paper, we see several examples of heading spaces. We can have an additional structure on *X*, but it is not used; only the multiplication of the elements of *G* by the elements of *X* is relevant. It is also possible to have a set *X* with some values that are not really used in the computation. If this happens, we consider in this section that the real heading space is the subset of *X* given by the orbits actually used in the computation with legitimate values.

It is always possible to have heading spaces for any number of orbits. If we want to have *n* orbits, we can use the heading space X=G×{1,2,⋯,n} with the action G×X→X given by $g\cdot \left(g^{\prime },i\right)=\left(gg^{\prime },i\right)$ for all $g,g^{\prime }\in G$ and all i∈{1,2,⋯,n}. It is also possible to use any other set *X* with an action for the group *G*.

**Definition** **1.** *A head is a pair (h,x) where x is in a heading space and h∈H. The value h is called the H-modifier of the head. The elements in the program is represented using heads, but the only visible element is the element x∈X. The H-modifier is known by the compiler or the programmer, but it is not visible during the computation.*

### 2.5. Correspondence between Heads and Elements of *M* (Private)

For every heading space *X*, we have a map $\pi_{X}:X\to M$ such that $\pi_{X}\left(gx\right)=g\pi_{X}\left(x\right)$ (a map between *G*-gets with this property is called a *G*-map).

We say that an element m∈M is represented by the head (h,x) if $m=h\pi_{X}\left(x\right)$. In this section, all the elements of *M* should be representable by heads for any heading space *X*; therefore, $\pi_{X}:X\to M$ should be surjective.

To get this surjectivity, we have to analyze the orbits of *M* and get a subset of representatives, $M_{0}\subseteq M$ such that $M=\cup_{m\in M_{0}}Gm$. For these elements *m*, we need to get values in *X* such that $\pi_{X}\left(x\right)=m$, but this cannot be done without some restrictions.

Let *m* be an element of $M_{0}$ and suppose x∈X satisfies $\pi_{X}\left(x\right)=m$. Let $G_{m}=\left\{g\in G:gm=m\right\}$ and $G_{x}=\left\{g\in G:gx=x\right\}$ be the stabilizers of these elements. The condition $\pi_{X}\left(x\right)=m$ implies that, for all $g\in G_{x}$, $g\pi_{X}\left(x\right)=\pi_{X}\left(gx\right)=\pi_{X}\left(x\right)=m$, therefore $g\in G_{m}$. This proves that the element x∈X that represents *m* should satisfy the condition for the stabilizers $G_{x}\subseteq G_{m}$. This is in fact the only condition required.

If we use a heading space of the type G×{1,2,⋯,n}, the values (1,i) always have a trivial stabilizer $G_{\left(1,i\right)}=\left\{1\right\}$ and this is contained in any possible stabilizer $G_{um}$. In this case, we can pick any index *i* to represent the elements of the orbits $G_{um}$ without restriction.

### 2.6. Base Points (Private)

We fix a set with one or more elements B⊆M. They are called base points.

**Definition** **2.** *A link is a triple (h,g,b)∈H×G×B. The value h is called the H-modifier of the link. The value hgb∈M is called the element represented by the link.*

Elements in the program are represented using heads and links. In the case of links, the only visible element in the program is the element g∈G; the values *h* and *b* are known only by the compiler or the programmer.

Not all the elements of *M* need to be representable using a single link. In fact, we use multiple links to represent the elements of *M*.

If we denote hGb={hgb∈M:g∈G}, we represent the elements in *M* as a sum of elements in the subsets hGb for h∈H and b∈B. Given a number of subsets, the elements of *M* that can be written as the sum of elements in the chosen subsets is called a sumset. This problem is well studied in additive number theory (see [12] for details), but, in our case, we have multiple solutions and a simple check lets us find them.

### 2.7. Reduction Maps (Public)

Let *X* and *Y* be heading spaces. A reduction map r:X→Y is a map for which we can find h∈H and b∈B such that

$$h\pi_{Y}\left(r\left(x\right)\right)=\pi_{X}\left(x\right)+b$$

for all x∈X. The pair (b,h) is called the type of the reduction.

These maps can always be constructed because we can take any x∈X, compute $h^{-1}\left(\pi_{X}\left(x\right)+b\right)\in M$ and find a value *y* such that $\pi_{Y}\left(y\right)=h^{-1}\left(\pi_{X}\left(x\right)+b\right)$. Any value *y* satisfying the condition can be defined as r(x).

Let r:X→Y and s:Y→Z be two reduction maps with such that the type of *r* is (b,h) and the type of *s* is (c,l). Then, we can make the composition of these reductions to have

$$h\left(l\pi_{Z}\left(s\left(r\left(x\right)\right)\right)\right)=h\left(\pi_{Y}\left(r\left(x\right)\right)+c\right)=h\pi_{Y}\left(r\left(x\right)\right)+hc=\pi_{X}\left(x\right)+b+hc.$$

This proves that the composition sr is actually a reduction map with type (b+hc,hl).

### 2.8. Dissolving Maps (Public)

A dissolving map for the heading space *X* with dimension *d* is a map

$$D:X\to G\times G\times \cdots \times G=G^{d}\;D\left(x\right)=\left(D_{1}\left(x\right),D_{2}\left(x\right),\cdots ,D_{d}\left(x\right)\right).$$

This map has hidden values $b_{1},b_{2},\cdots ,b_{d}\in B$ and $h_{1},h_{2},\cdots ,h_{d}\in H$ such that, for all x∈X,

$$\pi_{X}\left(x\right)=h_{1}D_{1}\left(x\right)b_{1}+h_{2}D_{2}\left(x\right)b_{2}+\cdots +h_{d}D_{d}\left(x\right)b_{d}.$$

The values $b_{i}$ and $h_{i}$ could be repeated.

The dissolving maps are public, but values $b_{i}$ and $h_{i}$ are private. The number of dissolving maps depends on the implementation; it could be possible in some cases to create a full implementation without dissolving maps. The value *d* is not fixed; we can have dissolving maps with different dimensions in the implementation.

The dissolving maps are used to transform heads into links. This is something that could be necessary during the computation. If we know by construction that this is only applied to a certain subset of heads, it is possible to define the map *D* only over this subset.

### 2.9. Control Values and Deals (Private)

All elements in *V* can be represented in many ways in terms of *M*. During the computation, it may be reasonable not to reuse the representations, especially in critical points. For example, suppose $V=F_{2}^{4}$, $M=F_{2}^{6}$, and *P* is an invertible matrix 6×6 over $Z_{2}$. We can define the projection p:M→V as the first four bits of Pm and consider the last two bits as control values.

If we use different control values at different critical points, we know that the representatives of the elements will never coincide.

There are many ways to define the control values. They can be checked before the *H*-modifiers or after them. It is also acceptable that the control values are not computed in the same way during the program. The multiple representation of the values and the way to control which representative is used during the computation is something that the programmer can use to increase the security level with great flexibility, but this is considered in Section 3.

A set of representatives that can represent all the elements of *V* but with a constant control value is called a deal. Using different deals at critical points is a way to protect against code injection.

### 2.10. The Program (Public)

Under white-box premises, the program is available to the attacker and, therefore, it can be considered as public information. The arithmetical elements in the program are a combination of heads and links. The number of heads and links used to represent a single element is dynamic and it changes during the execution of the program.

Each head or link has a visible part (the element x∈X or the element g∈G), and hidden parts, the *H*-modifier *h* and a base point b∈B for links. Thus, the element x∈X can represent the value $h\pi_{X}\left(x\right)$ for any h∈H and the link with value g∈G in the program, can represent any hgb for h∈H and b∈B. These hidden parts of the arithmetical element are known by the compiler or the programmer to decide which operations are possible and which tables have to be used at a particular moment. The heads can be in different heading spaces.

Suppose we have an element given by some heads $x_{i}$ and some links $g_{j}$, the real value could be $\sum_{i}h_{i}\pi_{X_{i}}\left(x_{i}\right)+\sum_{j}h_{j}^{\prime }g_{j}b_{j}$. The addition of two arithmetical elements is just the juxtaposition of the values (i.e. it requires no code). The problem is that, after a number of operations, the elements are huge and we need a way to reduce their sizes. This is done with the reduction maps.

Suppose we have a head x∈X and a link *g*. Internally, we know that they have the same *H*-modifier (for example l∈H) and we also know that the base point for the link is b∈B. Suppose  we have a reduction map *r* such that the associated partition of *X* is $\cup X_{i}$ and $x\in X_{i}$ with associated constants $h_{i}=h$ and $b_{i}=b$. In this case, we can proceed as follows:

$$l\pi_{X}\left(x\right)+lgb=lg\left(g^{-1}\pi_{X}\left(x\right)+gb\right)=lg\left(\pi_{X}\left(g^{-1}x\right)+b\right)=lgh\pi_{Y}\left(r\left(g^{-1}x\right)\right)$$

$$=l\varphi_{g}\left(h\right)g\pi_{Y}\left(r\left(g^{-1}x\right)\right)=l\varphi_{g}\left(h\right)\pi_{Y}\left(gr\left(g^{-1}x\right)\right).$$

This proves that the new head $gr(g^{-1}x)$ is a head representing the value $l\pi_{X}\left(x\right)+lgb$ with hidden *H*-modifier $l\varphi_{g}\left(h\right)$. This process lets us reduce the size of the arithmetical elements combining a head with a link if they have the correct hidden modifiers and we have the appropriate reduction map. If  this is not the case, the reduction is not possible.

One of the characteristics of this arithmetic is that the designer has a number of possible reductions and he has to decide the precise order of the reductions. Any attacker wishing to modify a value, or paste a part of the code in another part of the program, or interchange values, immediately gets many meaningless values. At the same time, this makes the generation of the code more difficult, but this is a problem that is not visible in the final result. To have some freedom, it is reasonable to have many reduction maps, although the number required is not excessively large.

The final code is several steps that, in the visible program, look like $gr(g^{-1}x)$ for g∈G and *x* in a heading space. This long sequence of values is combined with the values coming from the input tables or even constants.

The reduction that we have explained thus far lets us combine a head with a link, but we could need to add two heads. In that case, one of the heads has to be dissolved using a dissolving map into a number of links that let us continue the reduction process. This is not strictly necessary; depending on the design, it could be possible to have an implementation without dissolving maps. We see an example of this in Appendix B.

The designer of the program has to keep track of the *H*-modifiers for all possible executions of the program. This can be difficult, because the new *H*-modifier $\left(l\varphi_{g}\left(h\right)\right)$ depends on *g*, which is a value that could be input dependent. To deal with this problem, it is better to have a very simple function φ that could have at most two or three different values. This would let us classify the elements of *G* depending on the value $\varphi_{g}$ and keep track of the subset of *G* in which the values can range. If this is too complex for the implementation, a reasonable solution is to take a trivial map φ and therefore use the direct product H×G instead of a semi-direct product $H{⋊}_{\varphi }G$.

### 2.11. Input Tables (Public)

One of the critical points in a white-box implementation is the input of new values. The designer has to decide the correct representation that is used for the input values; they can be given by a head, a number of links or a combination of both. The control values let us use different deals in critical points. It is a good idea to use a deal for the input values that are not used at any other critical point of the program.

The *H*-modifiers of the input values are known by the designer, and the actual values given in the tables have to be checked to guarantee that all possible computations give the correct *H*-modifier in the following critical point. This is what we have mentioned in the previous subsection regarding the values $\varphi_{g}$ for all *g* in the input table.

### 2.12. Output Tables (Public)

This is the second critical point in the program. As we have done with the input values, the output uses a deal that has not been used at any other critical point. The *H*-modifiers of the elements when the output is reached should be known by the designer in order to build the table.

If we use a fixed control value for the output, the number of valid values is not large, thus we can create small output tables.

### 2.13. Tables for Nonlinear Operators (Public)

It is quite common to have nonlinear operators that have to be applied to the arithmetical operators. These are elements outside the arithmetic and we need a special table to define them. The  input and output of these tables are critical points of the implementation and, as we have done with the input and output tables, it is a good idea to use a unique deal (not used with any other critical point) to define the input entries and the output entries of this table. The tables are usually applied to heads and the output can be a head, a link or a combination of both.

### 2.14. Additional Affine Transformations (Private)

Consider the flow chart of the algorithm. The general structure is a graph with some critical points and a number of linear operations. Two points in the chart are said to be linearly equivalent if there are no critical points in between. This equivalence relation defines several connected components. All operations in a connected component are group operations, therefore we can apply any affine transformation to a whole connected component with the rule that everything that is done at the entry points of the connected component has to be undone at the exit points of the connected component. These operations can be glued to the tables at the critical points.

**Figure 1:**
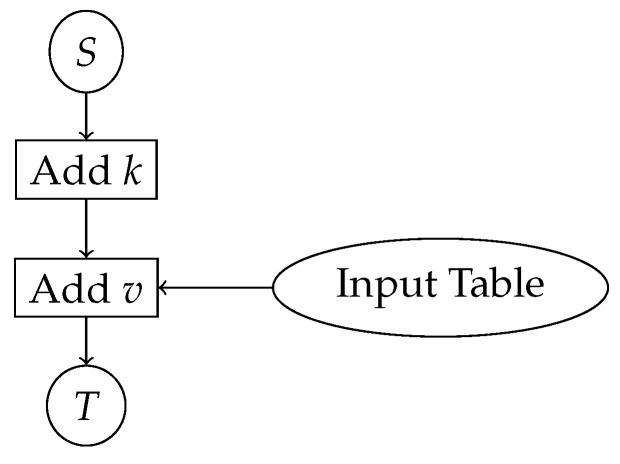
Example of a connected component.

Consider, for example, $V=Z_{7}$ and a program that computes a nonlinear operator *S*, adds a key value *k* and an input value *v*, and finally computes another operator *T* of the result. The graph of this connected component is in Figure 1.

Suppose that, by design, we decide that the output of *S* is multiplied by *a* and added to *b*. Instead of having *x*, we have ax+b and this has to be considered in the encoding of the input table, which should give us av instead of *v*. We can now add the value to the output of *S*, (ax+b)+av=a(x+v)+b. At the exit point of the connected component (the input of *T*), we have to undo the change and add the key *k*, thus the encoding of the table for *T* will compute $a^{-1}\left(y-b\right)+k$ before computing the real *T*. All these things can be included in the definition of the tables with no additional cost, but they are constant transformations that remain fixed for all executions of the  program.

## 3. Additional Security Measures

In Section 2, we are too strict in the definitions and properties required for a private arithmetic. This is a first approach and it is necessary to make a simplified version to introduce the theory. In this section, we are much more flexible. In a real implementation, it is important to include elements that could make the analysis more confusing to the attacker. The ideas of this section and others that could be considered by the programmer help to increase the security.

### 3.1. The Relation Between the Internal and the External Arithmetic

We have two commutative groups, *V* and *M*, and in Section 2 we have defined a projection p:M→V that lets us represent every element v∈V by at least one element m∈M. If we have to add two elements $v_{1}$ and $v_{2}$, we simply compute the sum of their representatives, and it is a representative of the sum because *p* is a homomorphism. However, this is not the only option. We could also have more than one element in *M* to represent the elements of *V*. Consider p:M×M×⋯×M→V a surjective group homomorphism and $v_{1},v_{2}\in V$ such that $p\left(m_{i}^{1},m_{i}^{2},\cdots ,m_{i}^{k}\right)=v_{i}$, then $v_{1}+v_{2}=p\left(m_{1}^{1}+m_{2}^{1},m_{1}^{2}+m_{2}^{2},\cdots ,m_{1}^{k}+m_{2}^{k}\right)$. When this happens, *V* is said to be a group generated by the group  *M*.

The most general construction is to have a surjective group homomorphism $p:M^{n}\to N$ such that *V* is a subgroup of *N*. We can emulate the arithmetic of the elements of *N* using tuples of elements in *M*, thus we can do it for *V* because all the elements of *V* are in *N*. When *V* is a subgroup of a group generated by *M*, it is said to be subgenerated by *M*.

The category of groups or modules subgenerated by *M* is denoted by σ[M] in the literature (see  [13], Chapter 3, Section 15). This category is closed under submodules, coproducts and quotients, therefore we cannot get any further groups iterating the constructions.

Making the group *M* quite different from *V* is something that increase the security, because the group *V* is public (it is usually described in the algorithm), but the group *M* and the relation between *M* and *V* is not known by the attacker, thus the internal arithmetic would be safe.

### 3.2. Quasi-Reductions

In Section 2.7, we show that the reductions can be composed. This property could be used by the attacker because, making all possible compositions, some patterns could be revealed. It is also usual to compare different reductions in order to remove some security measures (this is usually called differential cryptanalysis). For example, consider *X* and *Y* two heading spaces, r:X→Y a reduction with type (b,h) and $x_{1},x_{2}\in X$.

The type of *r* gives

$$h\pi_{Y}\left(r\left(x_{1}\right)\right)=\pi_{X}\left(x_{1}\right)+b\;h\pi_{Y}\left(r\left(x_{2}\right)\right)=\pi_{X}\left(x_{2}\right)+b.$$

If we subtract these values, we get

$$h\left(\pi_{Y}\left(r\left(x_{1}\right)\right)-\pi_{Y}\left(r\left(x_{2}\right)\right)\right)=\pi_{X}\left(x_{1}\right)-\pi_{X}\left(x_{2}\right).$$

This equation has removed the value *b* and introduced some equations that could be used by the  attacker.

The idea of quasi-reductions is to consider maps r:X→Y that do not satisfy the condition $h\pi_{Y}\left(r\left(x\right)\right)=\pi_{X}\left(x\right)+b$ for any (h,b) and mix them with real reduction maps to introduce a non-arithmetical behavior in the program that could make any attack based on arithmetical properties difficult, in particular, differential cryptanalysis. This property also avoids the possibility of  composition.

We show how to deal with these quasi-reductions with an example that is expanded in Section 5. Suppose $V=F_{2}^{8}$ and $M=F_{2}^{20}$. The projection p:M→V is given by the first eight bits and consider that the last four bits have a non-arithmetical behavior (let *E* be the subspace generated by these four bits).

A quasi-reduction r:X→Y satisfies $h\pi_{Y}\left(r\left(x\right)\right)=\pi_{X}\left(x\right)+b$ for the first 16 bits, but the last four bits have random values (or any other pattern that could be interesting to hide the real structure). If we have to compute the addition of two elements $m_{1}$ and $m_{2}$, we can be sure that the first 16 bits are actually the addition of the first 16 bits of $m_{1}$ and the first 16 bits of $m_{2}$, but the last four bits have a value fixed by the definition of *r*, but not following any addition rule. These extra values break the arithmetical analysis, thus, given two values $x_{1}$ and $x_{2}$ in *X*, we have

$$h\left(\pi_{Y}\left(r\left(x_{1}\right)\right)-\pi_{Y}\left(r\left(x_{2}\right)\right)\right)=\pi_{X}\left(x_{1}\right)-\pi_{X}\left(x_{2}\right)+e\left(x_{1}\right)-e\left(x_{2}\right).$$

where $e\left(x_{1}\right)-e\left(x_{2}\right)$ is an *error* that belongs to *E* and depends on $x_{1}$ and $x_{2}$ and, hence, it is quite difficult to analyze.

The main objective of this subspace *E* is to introduce values to break the arithmetical properties of the tables but we have to give correct results in spite of these errors. Therefore, we have to keep these values *under control*. The easiest way is that *E* would be a $k[H{⋊}_{\varphi }G]$-submodule of *M*. In this way, every element in *M* can be decomposed in two parts, the error e∈E and a value in M/E that behaves arithmetically (without errors). Space *E* is not visible, thus only the programmer can *clean* the errors from the values in order to generate correct computations.

### 3.3. Valid and Forbidden Elements

Consider an attacker trying to obtain information from the public elements. The attacker has a group *G* and elements in a heading space *X* that can be multiplied by elements in *G*, thus, given x∈G, it is quite natural to make all possible multiplications {gx:g∈G}. This is called the orbit of the action. These orbits over *X* are related with the orbits of the action G×M→M because the element *x* represents an element in *M*.

The elements of *M* can be multiplied by elements of *G* and elements of *H*. The elements of *H* modify the element to be represented using some fixed rules, therefore it is interesting to analyze the orbits of *M* as $H{⋊}_{\varphi }G$-set. The orbit of m∈M is $\left\{hgm:hg\in H{⋊}_{\varphi }G\right\}=\left(H{⋊}_{\varphi }G\right)m$.

Our objective is to use orbits that cannot be easily recognized, but some elements could be a problem. For example, the case of 0∈M is very particular. The elements of *H* and *G* are linear transformations, therefore hg0=0 for all h∈H and g∈G, thus 0 generates an orbit with only one element. We would prefer orbits not to be very different and this special orbit can be a problem. This can also be true for other special orbits. The idea is to remove these special elements from the arithmetic and use only *normal* elements. We analyze in a bit more depth why these elements appear.

First, note that the elements of *H* and *G* are endomorphisms of *M* and therefore have linear properties in the multiplication $(H{⋊}_{\varphi }G)\times M\to M$. This makes *M* not only an $H{⋊}_{\varphi }G$-set but also a *R*-module for the group ring $R=Z[H{⋊}_{\varphi }G]$. The commutative group *M* is thus a *R*-module and the composition series, $0=M_{0}\leq M_{1}\leq \cdots \leq M_{n}=M$, is unique up to equivalence (by Jordan–Hölder theorem). As we show, the orbit generated by $M_{0}$ only has one element, but it is possible to have small orbits in $M_{1}$ and even in $M_{2}$. The elements of these small orbits are better to be removed.

Let $\tilde{M}$ be a subset of elements in *M* that we want to remove from the arithmetic (they are called forbidden elements), and we have a reduction r:X→Y with type (b,h). The elements of $\tilde{M}$ can be removed from the image of $\pi_{Y}$ and $\pi_{X}$; however, what happens if we have x∈X such that $h^{-1}\left(\pi_{X}\left(x\right)+b\right)$ is a forbidden element? In that case, we cannot make the definition of r(x) and the compiler should ensure that this reduction is never going to be executed in the program. If r:X→Y is the only reduction with origin in *X*, this implies that $\pi_{X}\left(x\right)$ can also be considered a forbidden element for *X* and it can be included in $\tilde{M}$ although its orbit could be *normal*.

This shows that the forbidden elements could depend on the heading space *X* and the projection $\pi_{X}:X\to M$, which could be undefined, not only because $\pi_{X}\left(x\right)$ is a forbidden element but also because the reductions would take it to a forbidden element in another heading space, thus the family of forbidden elements is something dynamic. In Section 5, we show how to analyze the orbits and select the forbidden elements with an example.

### 3.4. Overlapping Orbits and Combinations of Heading Spaces

We show in previous subsections that the attacker can get some information by playing with the reductions and analyzing the orbits. The implementation should make this task as difficult as  possible.

A heading space is a set *X* with a multiplication by elements of *G*. It is not even necessary that all the multiplications give correct results, because they could correspond to forbidden elements. If  we have several heading spaces $X_{1},X_{2},\cdots ,X_{k}$, we can join them in a single set *X* as a disjoint union. If  *X* and *Y* are combinations of heading spaces, a reduction map r:X→Y is a combination of reduction maps.

For an attacker, it is not completely trivial to decide if two elements $x_{1},x_{2}\in X$ belong to the same heading space or not. It is even more difficult to know all the elements that belong to the same heading space. The attacker could use the following claims to group the elements that belong to the same heading space.

1. All elements that appear at the same position in the program under different valid inputs belong to the same heading space.
2. For every x∈X, all elements in the orbit of *x*, Gx, belong to the same heading space.
3. Let x∈X and r:X→Y be reduction maps, then all the values r(gx) belong to the same heading space for all g∈G.

The first claim is difficult to avoid, but only gives partial information because, at a certain point of the program, the elements not only belong to the same heading space, but also have some fixed control values; therefore, the subset of *X* obtained using this technique is only a small part of the heading  space.

The second claim can be very useful to incorporate errors into the analysis of the attacker. We  know that not all the elements of the orbit Gx are valid elements. Suppose that the elements not used in the orbit Gx are precisely those needed by other orbit in another heading space to represent their valid elements. Then, we can use the orbit Gx with two different meanings, depending on the value g∈G. We call this method overlapping orbits. It is not easy that this property happens by chance, but it could be possible if we select the orbits that belong to each heading space looking for this particular property. We show this in Section 4. Overlapping orbits is not easy, and the tables do not have more than a few of them overlapped, but these can be enough to introduce errors in the analysis of the attacker.

The third claim is not true. Several values of r(gx) are invalid and we can define the tables with any value we want. The values chosen for the invalid elements can be in any heading space. If  the orbit Gx has elements overlapped with another orbit, it is even worse because the values r(gx) are in different heading spaces and they are actually used in the computation.

## 4. Discussion

We list some of the most notable features of this method. Some of them are qualitative and others can be quantified.

### 4.1. Flexibility

A private arithmetic assumes that the external commutative group is fixed, but all other constructions can be decided. All the internal arithmetic can be decided by the programmer depending on the resources (time and memory) and his own expertise in automorphism groups.

The selection of the orbits, their representatives, the base points, etc. can be decided freely. The  number of choices is really high.

### 4.2. Non-Standard Operators

The most natural choice to obfuscate the addition in a commutative group is to have one or more binary operators to be applied over encrypted operands. The standard attacks try to match encrypted values with plain values and to use the algebraic properties of the group addition to find as many plain values as possible.

The method given in this paper has one or more reduction maps. The reduction maps are unary operators. The visible algorithm also provides a multiplication between elements in a group *G* and the heading spaces. The group *G* cannot be matched with elements in the external commutative group and the standard attacks do not fit with the arithmetical structure given by the reductions.

### 4.3. Nonlinear Relations Between Values and Their Representatives

The arithmetical elements in the program are represented by heads and links. The visible elements are elements in the heading space for heads and elements in *G* for links. The connection between these elements is not linear; for example, suppose $g_{1}$ and $g_{2}$ are the visible part of two links. Even if they would have the same *H*-modifier and the same base point, $hg_{1}b+hg_{2}b$ is in general not connected with any hgb for any g∈G, because *G* is closed under group multiplication, but the addition is not allowed. Something similar happens for heads. Consider the example given in Appendix B, two heads $x_{1},x_{2}$ could have visible representatives $v_{1},v_{2}\in Z_{10}^{2}$. The addition $v_{1}+v_{2}\in Z_{10}^{2}$ has no connection at all with the head that represents the addition of $\pi_{X}\left(x_{1}\right)+\pi_{X}\left(x_{2}\right)$

The nonlinear relation between values and their representatives is something that many obfuscations consider, but, in most cases, it is done in an artificial way, which must be undone before the obfuscated additions. In this system, the non-linearity is completely natural and fits the structure of the computations.

### 4.4. Linear Growth

We have previously mentioned that the arithmetical operators (the reductions) are unary operators instead of binary ones. This property means that the growth of the tables is linear, while the growth of the security elements is quadratic. For example, suppose that $M=F_{2}^{k}$. The heading spaces has a number of elements with the same order of magnitude of *M*, and we can say $O(2^{k})$. The number of invertible matrices k×k over $Z_{2}$ (which could be considered as a measure of the security level, as shown in Section 4.6) is $O\left({\left(2^{k}\right)}^{2}\right)=O\left(2^{2k}\right)$, a quadratic growth in comparison with the growth of the size of the tables. Using binary operators, the table would require two inputs and thus the growth of the tables would also be quadratic.

The linear growth lets us optimize the security for the resources available, increasing the size of *M* and getting a much greater increase in the security than when using binary operators. This property is especially relevant in the case of IoT because the resources are usually quite limited.

### 4.5. Protection against Differential Cryptanalysis

Another noteworthy characteristic of this system is that we can incorporate non-arithmetical behavior to the operators. This is what we have called quasi-reductions, and we explain them in Section 3.2. These quasi-reductions increase the protection against differential cryptanalysis.

### 4.6. Security and Conjugacy Search Problem

There are algorithms in which we can obtain the secret information only playing with the inputs and outputs. In that case, even a black-box implementation of the algorithm would reveal the secret information and it is worthless to make a white-box implementation that would not increase the security at all, because the attacker has a running implementation by definition of white-box. This is what we call intrinsic vulnerabilities and we show an example of this in Appendix B.1.

There are other vulnerabilities due to naive choices, for example, taking M=V, or a group *G* that generates orbits that can be easily analyzed.

There are also algorithms with many mathematical properties that are not evident at first sight and that can be used to get the secret information despite the encoding. Strictly speaking, these are not intrinsic vulnerabilities because they can be local to a piece of the code. For example, suppose that we have a program given in rounds, such that, if we randomly modify a value at the beginning of a round, this change is spread in a way that depends on the secret values. A blind obfuscation of the algorithm could make it easy to recognize the structure of rounds and independently of the arithmetical obfuscation, it is possible to modify a value at some point with the value given in a previous execution. This code injection combined with the mathematical property of the algorithm known by the attacker could reveal the secret information without actually breaking the arithmetic.

These vulnerabilities make it almost impossible for an automatic program to generate obfuscated code. There are even theoretical studies about the impossibility of having a general virtual black box system. See [14,15] for details.

The advanced encryption standard (AES) is a typical example of this. There are many publications studying the mathematical properties of this algorithm that can be used to break white-box implementations. In particular, AES is inherently vulnerable to differential fault analysis (DFA) and this kind of attacks can be applied to the majority of the public implementations of AES (see, for example, [2]). However, not only DFA, the number of known attacks to white-box implementations of AES is huge. The experience of the WhibOx Challenge (see [8]) shows that many implementations of AES can be broken independently of the arithmetical encoding.

The method presented in this paper helps to protect, mainly because it is possible to use multiple representations of the elements with different control codes, but not all possible design vulnerabilities can be avoided if the structure of the program is revealed in the obfuscated code, and this is something that the designer of the program has to decide, independently of the arithmetic.

What we can analyze here is the security related to the arithmetical elements. We use in this system a group *G* that is a subgroup of Aut(M). The natural choice is to have a *G* much smaller than Aut(M). For example, if *M* is $F_{2}^{20}$, Aut(M) is the set of invertible matrices 20×20 over $F_{2}$. The number of them is $\prod_{i=0^{19}}\left(2^{20}-2^{i}\right)\approx 2^{398.2}$. The group *G*, in comparison, is really small; in the example given in Section 5, it has 2520 elements.

In recent years, the number of cryptographic algorithms based on non-commutative groups has increased. We can see in [11] several algorithms based on non-commutative groups and the group-theoretical problems on which they are based. In the system proposed in this paper, we have a group *G* that it is given by a table or any other method that lets us compute with the elements. The  attacker knows that this group is a subgroup of Aut(M) but the internal group *M* is not known. Using the critical points, in particular the input and output tables, it could be possible to get some information about the properties of the values in the external group *V* compared to their corresponding internal values in *M*. This information is always partial. In the worst scenario, the attacker could guess that the original representation of the group *G* in Aut(M) is made using some special representation. However, this original representation ψ:G→Aut(M) can be modified up to conjugacy using any invertible element t∈Aut(M) with the equivalent representation $\psi^{\prime }\left(g\right)=t\psi \left(g\right)t^{-1}$. The  number of options for *t* can be huge and these new representations change completely the elements that belong to each orbit and their properties. The attacker needs to determine the conjugacy used with very short information about the effects in the external group. The conjugacy problems are considered hard to solve and the security of group-theoretic cryptography is very often based on them. See [11] for general background on these kinds of problems.

### 4.7. Use Cases and Further Applications

In the Introduction we give several use cases for white-box cryptography. In general, white-box cryptography should be considered when the attacker has access to the program and can manipulate it without restrictions to obtain the critical information, for example the keys.

As we can see in [16], the problem of mistrust in IoT is one of the main factors that have affected how IoT security is perceived. IoT devices can become adversaries themselves and they can be used to obtain critical information. The protection of our code with a white-box obfuscation makes this impossible, because the valuable information is not visible to the hostile environment.

The method proposed in this paper is not only valid in general as a white-box method. We show above that it is possible to generate a huge number of different arithmetics choosing some parameters, which makes it especially useful for the generation of code for IoT. Another characteristic of the code is the homogeneity. The code generated using this method has long sequences of reduction steps, each requiring a similar table lookup (we can see an example of this in Algorithm A3). The  white-box protection let us delegate the computation of a number of these reductions to other devices without risk, because the program is supposed to be public. As we can see in [17], the security is a major threat in edge and fog computing, but using this method it is possible to avoid that risk.

## 5. A Huge Private Arithmetic

In this section we analyze an example with all the security measures considered in the paper and a huge size. The arithmetic is generated for the group $V=F_{2}^{8}$. We also analyze the memory requirements to generate this arithmetic.

The internal commutative group *M* is $F_{2}^{20}$. The elements of *M* are decomposed infive subvectors of four bits each. Thus, we denote $m=(m_{0},m_{1},m_{2},m_{3},m_{4})$ the vector of *M* and the $m_{i}$ is vectors in $F_{2}^{4}$. We use the subvector $m_{4}$ as error space and the others as arithmetical values.

We use block matrices with five blocks of 4×4-matrices in the diagonal to generate *G*. The group of invertible 4×4-matrices over $F_{2}$ has 20,160 matrices and generates some orbits that are too big for our purposes. It is better to consider a subgroup of this group with 2520 elements and combine it with some permutations of the five blocks in the diagonal. In this case, we consider *H* the subgroup generated by the rotation of the first four blocks. This generates a group *H* with order four and the elements of *H* commute with the elements of *G*, thus we have a direct product instead of a semi-direct product for the extended arithmetical group H×G.

There are many combinations that let us generate the subgroup of 2520 elements. One of them is to use the matrices $a=\left[\begin{array}{cccc} 1 & 0 & 1 & 1\\ 0 & 1 & 0 & 0\\ 1 & 1 & 1 & 0\\ 0 & 0 & 1 & 1 \end{array}\right]$ and $b=\left[\begin{array}{cccc} 1 & 0 & 1 & 0\\ 1 & 1 & 0 & 0\\ 0 & 0 & 0 & 1\\ 0 & 1 & 0 & 0 \end{array}\right]$ in ${\mathrm{Mat}}_{4\times 4}\left(F_{2}\right)$. Many others can be found by conjugacy.

The group $G_{0}$ generated by these two matrices has 2520 elements. For each $g\in G_{0}$, we can make the block matrix $\left[\begin{array}{ccccc} g & 0 & 0 & 0 & 0\\ 0 & g & 0 & 0 & 0\\ 0 & 0 & g & 0 & 0\\ 0 & 0 & 0 & g & 0\\ 0 & 0 & 0 & 0 & g \end{array}\right]\in {\mathrm{Mat}}_{20\times 20}\left(F_{2}\right)$ that is identified with *g*. The matrix *h* to generate *H* could be the block matrix $h=\left[\begin{array}{ccccc} 0 & 0 & 0 & I & 0\\ I & 0 & 0 & 0 & 0\\ 0 & I & 0 & 0 & 0\\ 0 & 0 & I & 0 & 0\\ 0 & 0 & 0 & 0 & I \end{array}\right]\in {\mathrm{Mat}}_{20\times 20}\left(F_{2}\right)$, where *I* is the identity matrix in ${\mathrm{Mat}}_{4\times 4}\left(F_{2}\right)$.

At this point, it is necessary to decide which elements of *M* that are valid. This  decision requires some analysis of the group ring and the orbits because our intention is to remove the elements that could have a recognizable behavior in the tables. We have to analyze the Z[G] structure of *M*. It is equivalent to use this structure or the $F_{2}\left[G\right]$-structure because *m* has characteristic two, thus we can take R=Z[G] or $R=F_{2}\left[G\right]$.

The *R*-module *M* has a composition series $0=M_{0}\leq M_{1}\leq M_{2}\leq M_{3}\leq M_{4}\leq M_{5}=M$, in which $M_{i+1}/M_{i}$ are simple *R*-modules. The composition series in this case has $M_{i+1}/M_{i}$ always isomorphic to $F_{2}^{4}$, the vector space of 16 elements with the structure given by $G_{0}\times F_{2}^{4}\to F_{2}^{4}$ (note that $G_{0}$ and *G* are isomorphic). The Jordan–Hölder theorem says that any other composition series $0=M_{0}^{\prime }\leq M_{1}^{\prime }\leq M_{2}^{\prime }\leq M_{3}^{\prime }\leq M_{4}^{\prime }\leq M_{5}^{\prime }=M$ has the same length and $M_{i+1}^{\prime }/M_{i}^{\prime }$ is also isomorphic to the same simple module.

We use a very simple decomposition in blocks that makes the structure visible. The structure (up to isomorphism) is not changed if we consider a conjugated action given by $t^{-1}Gt$ with any invertible matrix *t*. It is better to make the theoretical analysis with the original version and apply the conjugation afterwards.

It is an obvious choice to remove the element 0, but we also remove the elements of the different *R*-modules that can appear in the first two steps. In the version before conjugation, the elements $(m_{0},m_{1},m_{2},m_{3},m_{4})\in M$ such that they can appear in the position $M_{1}$ in the composition series are the ones such that $\dim(\mathrm{span}\left(m_{0},m_{1},m_{2},m_{3},m_{4}\right))\leq 1$, and the ones that can appear in the position $M_{2}$ are the ones such that $\dim(\mathrm{span}\left(m_{0},m_{1},m_{2},m_{3},m_{4}\right))\leq 2$. If we want to consider them as forbidden elements, we have to restrict the subset of valid elements to those ones such that $\dim(\mathrm{span}\left(m_{0},m_{1},m_{2},m_{3},m_{4}\right))\geq 3$ or a subset of these elements. There are 1,015,560 elements satisfying this condition. They are distributed in 403 orbits with 2520 elements per orbit.

They can be classified using matrices 4×5 with columns $\left(m_{0},m_{1},m_{2},m_{3},m_{4}\right)$ and defined normal forms for the generator of the orbits. Consider a map f:{0,1,2}→{0,1,2,3,4} strictly increasing. We fix in the vectors $\left[\begin{array}{c} 1\\ 0\\ 0\\ 0 \end{array}\right]$, $\left[\begin{array}{c} 0\\ 1\\ 0\\ 0 \end{array}\right]$ and $\left[\begin{array}{c} 0\\ 0\\ 1\\ 0 \end{array}\right]$ in the positions f(0), f(1) and f(2). The rest of the values will be free if they keep the matrix in an almost echelon form. Strictly speaking, it is not an echelon form because the group can reduce only three positions (it is not the full group of invertible matrices). The generators are given in Table 1.

For each heading space, we have to choose which elements are used. We have to be careful to have multiple representations for each element in *V*, but we can choose the orbits we want. We could decide to have heading spaces with a number of orbits between 200 and 300 from these 403 ones. The  selection of these orbits can be made in order to get properties such as overlapping orbits (see Section 3.4). For example, consider *X* and $X^{\prime }$ as two heading spaces that have an orbit (G,i) overlapped. We define at the same time two reductions r:X→Y and $r^{\prime }:X^{\prime }\to Y^{\prime }$. The types of the reductions *r* and $r^{\prime }$ are (h,b) and $\left(h^{\prime },b^{\prime }\right)$. We select a value $x_{i}\in X$ and $x_{i}^{\prime }\in X^{\prime }$ to be the base point on these orbits and finally we compute $u_{g}:=h^{-1}\left(gx_{i}+b\right)$, $v_{g}:={\left(h^{\prime }\right)}^{-1}\left(gx_{i}^{\prime }+b^{\prime }\right)$ for all g∈G. The values $u_{g}$ and $v_{g}$ will belong to some orbits, the idea is to choose the orbits in *Y* and $Y^{\prime }$ in such a way that $u_{g}$ and $v_{g}$ are not valid elements in *Y* and $Y^{\prime }$ at the same time. The rest of the orbits can be chosen randomly, bearing in mind that some of them are now forbidden to have a correct definition of *r* and $r^{\prime }$ in the overlapped orbit (G,i). The forbidden elements in the orbits are filled in tables *r* and $r^{\prime }$ with fake values that can be part of different heading spaces, as explained in Section 3.4.

Let $k_{1},k_{2},\cdots ,k_{n}$ be the number of orbits for each heading space. If they are between 200 and 300, we can consider that the number of elements in the combined heading space will be around 250n. The  representation of each element is an index *i* for the orbit (two bytes) and two other bytes for the group element g∈G. The size of the tables required for these combinations of heading spaces and reductions are 2520·250·n·4 bytes, that is 2.5n megabytes where *n* is the number of heading spaces. A big implementation could use eight heading spaces, and therefore a number around 200 megabytes would be reasonable.

The rest of the implementation would require tables for input and output, and also for possible S-boxes, another 200 megabytes could be required and the code depends on the algorithm, but could be another 100 megabytes. If we put together all these numbers, the size of a big implementation would be around 500 megabytes.

If the attacker knows the details of this construction, the security would be based on the choice of an invertible matrix *t* such that the actual group *G* would be $tGt^{-1}$. The number of choices for the non-arithmetical part is the number of maps E→E. They do not even have to be bijective, thus the number is $16^{16}=2^{64}$. The number of choices for *t* is $\prod_{i=0}^{15}\left(2^{16}-2^{i}\right)\approx 2^{254.2}$. The conjugation by *t* induces a new relation between *M* and *V* as well as a change in the base points used to represent the classes. The search for the conjugacy matrix is much more difficult than the standard conjugacy search problem, because only partial information is visible.

## 6. Conclusions

White box implementations are not a simple task. The protection of the arithmetic is something that requires a lot of resources, which might not be available (especially on IoT applications), therefore it is critical to decide which are the elements that should be protected. The method presented in this paper can be used to obfuscate the arithmetic of a commutative group using a related non-commutative group. The idea of the paper is to completely shuffle the additive structure and use the endomorphisms to make computations.

Non-commutative groups are attracting the attention of the cryptographic community because there are many hard problems related to them that can be used for cryptographic purposes. The  method presented in this paper is very flexible and it is possible to modify the implementations by conjugation. This makes it possible to generate a huge amount of essentially different implementations based on the same original groups. The search for this conjugacy is not the only problem that the attacker has to solve, because many different choices can be made to increase the difficulty of the analysis.

## 7. Patents

The contents of this paper may be related to the following patent applications: WO2018015325 and WO2018115143.

## Figures and Tables

**Table 1:** Generators and number of orbits.

*f*	Generators	Number of Orbits
(0,1,2)	$\left[\begin{array}{ccccc} 1 & 0 & 0 & * & *\\ 0 & 1 & 0 & * & *\\ 0 & 0 & 1 & * & *\\ 0 & 0 & 0 & * & * \end{array}\right]$	$2^{8}=256$
(0,1,3)	$\left[\begin{array}{ccccc} 1 & 0 & * & 0 & *\\ 0 & 1 & * & 0 & *\\ 0 & 0 & 0 & 1 & *\\ 0 & 0 & 0 & 0 & * \end{array}\right]$	$2^{6}=64$
(0,2,3)	$\left[\begin{array}{ccccc} 1 & * & 0 & 0 & *\\ 0 & 0 & 1 & 0 & *\\ 0 & 0 & 0 & 1 & *\\ 0 & 0 & 0 & 0 & * \end{array}\right]$	$2^{5}=32$
(1,2,3)	$\left[\begin{array}{ccccc} 0 & 1 & 0 & 0 & *\\ 0 & 0 & 1 & 0 & *\\ 0 & 0 & 0 & 1 & *\\ 0 & 0 & 0 & 0 & * \end{array}\right]$	$2^{4}=16$
(0,1,4)	$\left[\begin{array}{ccccc} 1 & 0 & * & * & 0\\ 0 & 1 & * & * & 0\\ 0 & 0 & 0 & 0 & 1\\ 0 & 0 & 0 & 0 & 0 \end{array}\right]$	$2^{4}=16$
(0,2,4)	$\left[\begin{array}{ccccc} 1 & * & 0 & * & 0\\ 0 & 0 & 1 & * & 0\\ 0 & 0 & 0 & 0 & 1\\ 0 & 0 & 0 & 0 & 0 \end{array}\right]$	$2^{3}=8$
(0,3,4)	$\left[\begin{array}{ccccc} 1 & * & * & 0 & 0\\ 0 & 0 & 0 & 1 & 0\\ 0 & 0 & 0 & 0 & 1\\ 0 & 0 & 0 & 0 & 0 \end{array}\right]$	$2^{2}=4$
(1,2,4)	$\left[\begin{array}{ccccc} 0 & 1 & 0 & * & 0\\ 0 & 0 & 1 & * & 0\\ 0 & 0 & 0 & 0 & 1\\ 0 & 0 & 0 & 0 & 0 \end{array}\right]$	$2^{2}=4$
(1,3,4)	$\left[\begin{array}{ccccc} 0 & 1 & * & 0 & 0\\ 0 & 0 & 0 & 1 & 0\\ 0 & 0 & 0 & 0 & 1\\ 0 & 0 & 0 & 0 & 0 \end{array}\right]$	$2^{1}=2$
(2,3,4)	$\left[\begin{array}{ccccc} 0 & 0 & 1 & 0 & 0\\ 0 & 0 & 0 & 1 & 0\\ 0 & 0 & 0 & 0 & 1\\ 0 & 0 & 0 & 0 & 0 \end{array}\right]$	$2^{0}=1$
	Total	403

**Figure A1:**
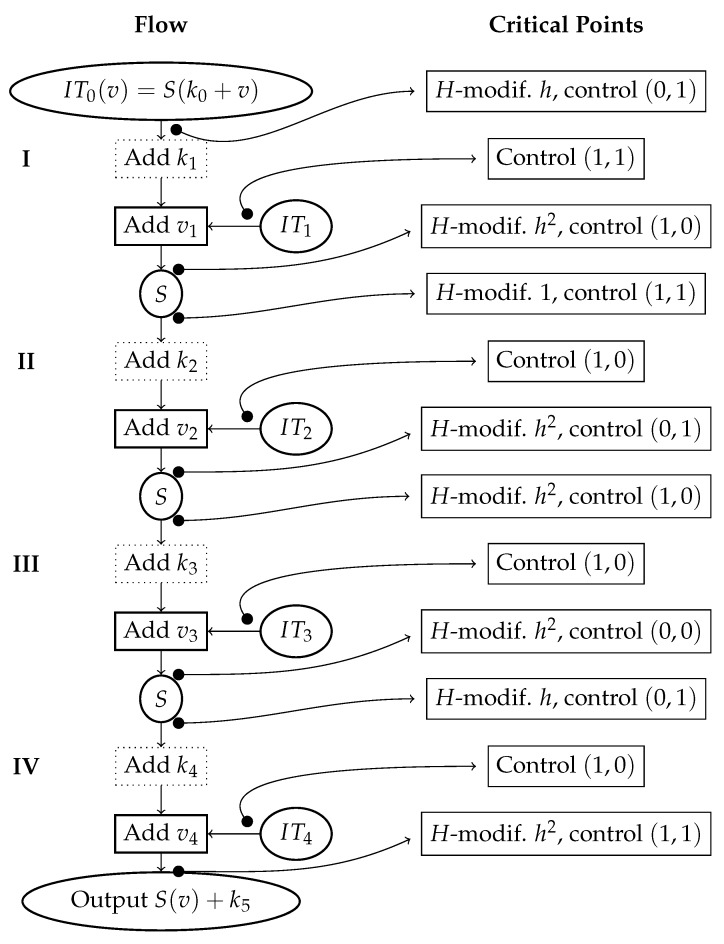
Connected components, flow and critical points.

## Abbreviations

   The following abbreviations are used in this manuscript:

DITEC	Departamento de Ingeniería y Tecnología de Computadores
DRM	Digital Rights Management
IoT	Internet of Things
AES	Advanced Encryption Standard
DES	Data Encryption Standard
CSP	Conjugacy Search Problem

## Appendix A. Summary of Mathematical Concepts and Notations

This appendix contains some standard mathematical definitions and notations used in this paper. For further references we suggest the book by Bhattacharya et al. ([18], Chapters 1–14) for basic definitions, the book by Wisbauer ([13], Sections 1–6) for rings and modules and the books by Lam [19] and Aschbacher [20] for group representations and more advanced results on group theory and connections with rings and modules.

**Group**	A set *G* with an operation ·:G×G→G that is associative. There exists an element 1∈G such that 1g=g1=g for all g∈G and for every g∈G there exists $g^{-1}\in G$ such that $g\cdot g^{-1}=1=g^{-1}\cdot g$.
**Commutative Group**	A set *M* with an operation +:M×M→M that is associative and commutative. There exists an element 0∈M such that 0+m=m+0=m for all m∈M and for every m∈M there exists −m∈M such that m+(−m)=(−m)+m=0.
**Ring**	A set *R* with two operations +,·:R×R→R and two special elements 0,1∈R such that (R,+,0) is a commutative group, the multiplication · is associative, r·1=1·r for all r∈R, and r(s+t)=rs+rt for all r,s,t∈R.
**Commutative Ring**	A ring *R* is said to be commutative if the multiplication is commutative. Examples are Z, the ring of integers and $Z_{n}$ the ring of modular integers for any integer n>1.
**Field**	A commutative ring in with every nonzero element *r* there exists $r^{-1}\in R$ such that $r\cdot r^{-1}=1$. Examples of fields are the rationals and the modular integers $Z_{p}$ when *p* is a prime number. If *p* is a prime, the field $Z_{p}$ can be denoted $F_{p}$. For instance, $Z_{2}$ and $F_{2}$ are exactly the same thing.
**Module**	Let *R* be a ring and *M* be a commutative group, we say that *M* is a module over the ring *R* if we have a multiplication ·:R×M→M such that (r+s)m=rm+sm, r(m+n)=rm+rn, 1m=m and (rs)m=r(sm) for all r,s∈R and m,n∈M. Every commutative group *M* is, in particular, a module over Z using the multiplication rm=m+m+⋯+m (*r*-times) and (−r)m=r(−m) for any integer r≥0 and m∈M. If *R* is a field, the modules over *R* are the vector spaces.
**Group Homomorphism**	Let *G* and *H* be groups. A map f:G→H is said to be a group homomorphism if f(k·g)=f(k)·f(g)for all k,g∈G and f(1)=1. If the groups are commutative, a homomorphism f:M→N should satisfy f(m+k)=f(m)+f(k) for all m,k∈M and f(0)=0.
**Group Isomorphism**	A group homomorphism *f* is said to be a group isomorphism if the map *f* is bijective. The inverse map will also be a group isomorphism.
**Group Endomorphism**	A group homomorphism between the group and itself is called an endomorphism.
**Group Automorphism**	A bijective group homomorphism is called automorphism. The set of group automorphisms is itself a group with the composition and the identity map as 1. Let *p* be a prime number and $Z_{p}$ the field of modular integers. The group of invertible matrices over $Z_{p}$ is an example of an automorphism group, because the automorphisms of $Z_{p}\times Z_{p}\times \cdots \times Z_{p}$ can be identified with the invertible square matrices.
**Module Homomorphism**	Let *M* and *N* be modules over *R* and f:M→N a group homomorphism considered as abelian groups. We say that *f* is a module homomorphism if f(rm)=rf(m) for all r∈R and m∈M. Using this additional property, we can define module isomorphisms, endomorphisms and automorphisms. If R=Z, the module homomorphisms and group homomorphisms are the same thing because f(rm)=f(m+m+⋯+m)=f(m)+f(m)+⋯+f(m)=rf(m). If *R* is a field, the module homomorphisms are the usual linear maps that can be identified with the matrices over the field.
**Endomorphism Ring**	Let *M* be a module over the ring *R*. The set of endomorphisms of *M* can be endowed with a ring structure if we define the addition of two endomorphisms f+g as (f+g)(m)=f(m)+g(m) for all m∈M. If *R* is a field, this ring is the ring of square matrices. If R=Z or any other ring, the endomorphisms are the natural generalization of matrices and play a similar role.
**Direct Product**	Let *H* and *G* be groups, the set H×G can be endowed with a group structure using the multiplication $\left(h,g\right)\left(h^{\prime },g^{\prime }\right)=\left(hh^{\prime },gg^{\prime }\right)$. With this structure the identity element in H×G is (1,1). It is straightforward to identify *G* with the elements {(g,1):g∈G} and *H* with {(1,h):h∈H}. In this way, the direct product can be written as HG and the elements as products hg with the rule that, although the multiplication in *H* and *G* might be non-commutative, the elements of *H* commute with the elements of *G*, i.e. gh=hg for all g∈G and h∈H.
**Semi-direct Product**	Let *H* and *G* be groups and φ:G→Aut(H) be a group homomorphism. We denote with $\varphi_{g}:H\to H$ the automorphism induced by *g* via φ. The semi-direct product of these groups, denoted by $H{⋊}_{\varphi }G$, is given by the pairs (h,g) with h∈H and g∈G endowed with the product $\left(h,g\right)\left(h^{\prime },g^{\prime }\right)=\left(h\varphi_{g}\left(h^{\prime }\right),gg^{\prime }\right)$. As we can see, the homomorphism φ acts as a modifier for the product in *H*. The direct product is a special case of this construction using the trivial homomorphism φ:G→Aut(H) such that $\varphi_{g}={\mathrm{id}}_{H}$ for all g∈G. Semi-direct products are a very common construction in group theory to build non-commutative groups, for example, the dihedral groups.
**Action of a Group**	Let *G* be a group and *X* be a set. An action of *G* over *X* is a map ·:G×X→X such that 1·x=x for all *x* and g·(h·x)=(g·h)x for all g,h∈G and all x∈X. A set *X* with an action G×X→X is called a *G*-set.
**Orbits and Stabilizers**	If we have an action of *G* on *X*, the orbit of an element x∈G is the subset of *X* given by {gx:g∈G}=Gx. The stabilizer of the element *x* is the subgroup of *G* given by the elements that fix *x*, $G_{x}=\left\{g\in G:gx=x\right\}$. The number of elements of $G_{x}$ is a divisor of the number of elements of *G* (by Lagrange’s theorem) and the number of elements in the orbit of *x* is $\left|G|/\right|G_{x}|$ (see ([18], Chapter 5, Theorem 4.7)).
**Group Ring**	Let *G* be a finite group and *R* be a ring. The group ring, denoted R[G] or RG, is the set of mappings u:G→R with the multiplication given by the rule $\left(u\cdot v\right)\left(h\right)=\sum_{g\in G}u\left(g\right)v\left(g^{-1}h\right)$. The standard notation for group rings is to identify the map u:G→R with the formal sum $\sum_{g\in G}u\left(g\right)\cdot g$ and multiply using the standard rules (r·g)(s·h)=(rs)·(gh) for all r,s∈R and g,h∈G.

## Appendix B. Full Detailed Example

In this appendix, we present a small example of the obfuscation of a cryptographic algorithm with the main elements that are commonly used in block ciphers and hash functions, in particular XOR operations and nonlinear permutations combined in several rounds. The example is developed in full detail, including all the tables required for the arithmetic. This requirement makes the example not completely realistic because the tables are much smaller than the ones used in a computer program. The idea of this appendix is to help the reader to reproduce the constructions with a guided example.

In this example, the arithmetic is over the vector space $V=F_{2}^{4}$, which is the external commutative group. The nonlinear permutation is the first one given in the data encryption standard algorithm

S=(14,4,13,1,2,15,11,8,3,10,6,12,5,9,0,7)

and the algorithm computes the function

$$\begin{array}{cccc} \xi : & V\times V\times V\times V\times V & \to & V\\ & \left(v_{0},v_{1},v_{2},v_{3},v_{4}\right) & \mapsto & \xi (v_{0},v_{1},v_{2},v_{3},v_{4}). \end{array}$$

The computation combines these values with six hidden keys $k_{0},\cdots ,k_{5}\in V$ using rounds. These values are the secret information that has to be protected.

**Algorithm A1** Example (plain version).
1: $w_{1}\leftarrow S\left(k_{0}⊕v_{0}\right)$ 2: $w_{2}\leftarrow S\left(k_{1}⊕v_{1}⊕w_{1}\right)$ 3: $w_{3}\leftarrow S\left(k_{2}⊕v_{2}⊕w_{2}\right)$ 4: $w_{4}\leftarrow S\left(k_{3}⊕v_{3}⊕w_{3}\right)$ 5: $w_{5}\leftarrow S\left(k_{4}⊕v_{4}⊕w_{4}\right)$ 6: **return** $k_{5}⊕w_{5}$

### Appendix B.1. Intrinsic Vulnerabilities

The first thing that we do with this example is to prove that it is possible to get the key playing with some inputs and outputs, independently of the implementation of the algorithm. This is what we call *intrinsic vulnerabilities*. Some algorithms reveal information regardless of the implementation. When this happens, it is impossible to get a secure version under the white box premises, because even a black box version of the program would be insecure. In this example, the reason is that the function ξ is too small but we make a white-box version of this function just to show the method, although we know that it is too small to be realistic.

The first and most evident reason this function is insecure is because the number of possible keys is $16^{6}=16777216$. This key space is small enough to be analyzed just by checking all the possibilities with a simple PC.

However, here, we give an attack that uses the nonlinearity of *S* to break the security. This vulnerability is not a special property of the permutation *S* that we have chosen. This is something general that can be done for all nonlinear permutations. Linear permutations do not have this vulnerability, but they have many others. Therefore, we cannot get rid of the nonlinearity, because it is a property required for the general security of the algorithms.

As a question of notation, we write the elements of $V=F_{2}^{4}$ using a single hexadecimal value. Thus, for example, the value b, that in binary form is 1010 represents the vector (1,0,1,0).

We use the auxiliary function f(v,u,k)=k⊕S(v⊕u) and the associated functions $g_{1}\left(u\right)=f\left(0,u,k\right)⊕f\left(3,u,k\right)$ and $g_{2}\left(u\right)=f\left(4,u,k\right)⊕f\left(a,u,k\right)$. The functions $g_{1}$ and $g_{2}$ do not depend on *k* because this value is canceled because k⊕k=0. If we compute $g_{1}\left(u\right)$ and $g_{2}\left(u\right)$ for all possible values *u*, we get that the possible outputs for $g_{1}$ are {f,9,a,4,c,2} and the outputs for $g_{2}$ are {4,3,8,2,e}. If we know the values $g_{1}\left(u\right)$ and $g_{2}\left(u\right)$ for a hidden *u*, we can obtain the value *u* looking at the following table

$$\begin{array}{ccccccc} & f & 9 & a & 4 & c & 2\\ 4 & 0 & e & * & * & * & *\\ 3 & b & 1 & * & 5 & * & f\\ 8 & 8 & 2 & 7 & 6 & 9 & c\\ 2 & 3 & d & * & * & * & *\\ e & * & * & 4 & * & a & * \end{array}$$

The values * are given to inputs that cannot be reached for any correct computation of the functions.

Now, we take the black-boxed version of ξ and fix the inputs $v_{0},v_{1},v_{2}$ and $v_{3}$. These values produce a value $w_{4}$ after the first 4 rounds. We want to compute the hidden value $u=k_{4}⊕w_{4}$, and, to do that, we see that $f\left(v,u,k_{5}\right)=\xi \left(v_{0},v_{1},v_{2},v_{3},v\right)$ is precisely $k_{5}⊕S\left(v⊕u\right)$ and we can compute the auxiliary values $g_{1}\left(u\right)=\xi \left(v_{0},v_{1},v_{2},v_{3},0\right)⊕\xi \left(v_{0},v_{1},v_{2},v_{3},3\right)$ and $g_{2}\left(u\right)=\xi \left(v_{0},v_{1},v_{2},v_{3},3\right)⊕\xi \left(v_{0},v_{1},v_{2},v_{3},4\right)$. From the previous table, we get the value *u*. Now, we only need to compute $\xi \left(v_{0},v_{1},v_{2},v_{3},0\right)=k_{5}⊕S\left(u\right)$ and recover $k_{5}$ because $k_{5}=\xi \left(v_{0},v_{1},v_{2},v_{3},0\right)⊕S\left(u\right)$. This technique has obtained the last key and reduced the problem to the function $\xi^{\prime }\left(v_{0},v_{1},v_{2},v_{3}\right)=S^{-1}\left(k_{5}⊕\xi \left(v_{1},v_{2},v_{3},v_{4},0\right)\right)$. This function has exactly the same structure but with one round fewer. We can apply the same technique to get the key $k_{4}$ and afterwards $k_{3}$, $k_{2}$, $k_{1}$ and $k_{0}$ by removing the security layers of this algorithm one by one.

A full explanation of the reasons for choosing these particular parameters 0,3,4 and a is outside the scope of this paper. It is a general technique to break encodings that is much more general and based on differential cryptanalysis. It is especially powerful on block ciphers and there are many publications on the topic. One general book to learn about these techniques is [21].

The main ideas that should remain after this subsection is that the example is merely to show how the arithmetic works with something small and we do not claim that the obfuscated version of this function is secure, because even the black-box version is insecure. Any algorithm can introduce intrinsic vulnerabilities that have to be studied in detail before starting a white-box implementation.

### Appendix B.2. Obfuscated Version (Public Elements)

In this subsection, we see a white-box implementation of the function ξ using the techniques explained in this paper. First, we show the public elements that are visible in the program and we make a simple computation. In the following subsection, we give the private elements of the implementation.

Apart for the external commutative group $V=F_{2}^{4}$ that it is part of the public algorithm, the first public element of this obfuscated implementation is the public group *G*. This group can be given by its multiplication table (Table A1). The structure of the group up to isomorphisms can also be considered public, because it can be easily computed using the multiplication table. The group *G* can be represented using endomorphisms over multiple commutative groups. For example, we can represent this group using matrices 2×2 over the rings $Z_{10}$ and also over $Z_{11}$. The correspondence between the element of the group and the matrices is given in Table A2. We include in this table the inverses of the elements in the group that are used during the computation. The value X is used to represent the number 10 in the ring $Z_{11}$.

**Table A1:** Group operation.

	$g_{0}$	$g_{1}$	$g_{2}$	$g_{3}$	$g_{4}$	$g_{5}$	$g_{6}$	$g_{7}$	$g_{8}$	$g_{9}$	$g_{10}$	$g_{11}$
$g_{0}$	$g_{0}$	$g_{1}$	$g_{2}$	$g_{3}$	$g_{4}$	$g_{5}$	$g_{6}$	$g_{7}$	$g_{8}$	$g_{9}$	$g_{10}$	$g_{11}$
$g_{1}$	$g_{1}$	$g_{0}$	$g_{5}$	$g_{6}$	$g_{7}$	$g_{2}$	$g_{3}$	$g_{4}$	$g_{10}$	$g_{11}$	$g_{8}$	$g_{9}$
$g_{2}$	$g_{2}$	$g_{5}$	$g_{0}$	$g_{9}$	$g_{8}$	$g_{1}$	$g_{11}$	$g_{10}$	$g_{4}$	$g_{3}$	$g_{7}$	$g_{6}$
$g_{3}$	$g_{3}$	$g_{8}$	$g_{7}$	$g_{10}$	$g_{5}$	$g_{11}$	$g_{1}$	$g_{9}$	$g_{6}$	$g_{2}$	$g_{0}$	$g_{4}$
$g_{4}$	$g_{4}$	$g_{9}$	$g_{6}$	$g_{5}$	$g_{3}$	$g_{10}$	$g_{7}$	$g_{1}$	$g_{2}$	$g_{8}$	$g_{11}$	$g_{0}$
$g_{5}$	$g_{5}$	$g_{2}$	$g_{1}$	$g_{11}$	$g_{10}$	$g_{0}$	$g_{9}$	$g_{8}$	$g_{7}$	$g_{6}$	$g_{4}$	$g_{3}$
$g_{6}$	$g_{6}$	$g_{10}$	$g_{4}$	$g_{8}$	$g_{2}$	$g_{9}$	$g_{0}$	$g_{11}$	$g_{3}$	$g_{5}$	$g_{1}$	$g_{7}$
$g_{7}$	$g_{7}$	$g_{11}$	$g_{3}$	$g_{2}$	$g_{6}$	$g_{8}$	$g_{4}$	$g_{0}$	$g_{5}$	$g_{10}$	$g_{9}$	$g_{1}$
$g_{8}$	$g_{8}$	$g_{3}$	$g_{11}$	$g_{1}$	$g_{9}$	$g_{7}$	$g_{10}$	$g_{5}$	$g_{0}$	$g_{4}$	$g_{6}$	$g_{2}$
$g_{9}$	$g_{9}$	$g_{4}$	$g_{10}$	$g_{7}$	$g_{1}$	$g_{6}$	$g_{5}$	$g_{3}$	$g_{11}$	$g_{0}$	$g_{2}$	$g_{8}$
$g_{10}$	$g_{10}$	$g_{6}$	$g_{9}$	$g_{0}$	$g_{11}$	$g_{4}$	$g_{8}$	$g_{2}$	$g_{1}$	$g_{7}$	$g_{3}$	$g_{5}$
$g_{11}$	$g_{11}$	$g_{7}$	$g_{8}$	$g_{4}$	$g_{0}$	$g_{3}$	$g_{2}$	$g_{6}$	$g_{9}$	$g_{1}$	$g_{5}$	$g_{10}$

**Table A2:** Group representations.

Group	$\mathrm{End}(Z_{10}^{2})$	$\mathrm{End}(Z_{11}^{2})$	Inverse
$g_{0}$	$\left[\begin{array}{cc} 1 & 0\\ 0 & 1 \end{array}\right]$	$\left[\begin{array}{cc} 1 & 0\\ 0 & 1 \end{array}\right]$	$g_{0}$
$g_{1}$	$\left[\begin{array}{cc} 9 & 7\\ 0 & 1 \end{array}\right]$	$\left[\begin{array}{cc} X & 2\\ 0 & 1 \end{array}\right]$	$g_{1}$
$g_{2}$	$\left[\begin{array}{cc} 1 & 3\\ 0 & 9 \end{array}\right]$	$\left[\begin{array}{cc} 1 & 9\\ 0 & X \end{array}\right]$	$g_{2}$
$g_{3}$	$\left[\begin{array}{cc} 9 & 2\\ 2 & 5 \end{array}\right]$	$\left[\begin{array}{cc} 6 & 1\\ 1 & 4 \end{array}\right]$	$g_{10}$
$g_{4}$	$\left[\begin{array}{cc} 5 & 2\\ 2 & 1 \end{array}\right]$	$\left[\begin{array}{cc} 7 & 1\\ 1 & 5 \end{array}\right]$	$g_{11}$
$g_{5}$	$\left[\begin{array}{cc} 9 & 0\\ 0 & 9 \end{array}\right]$	$\left[\begin{array}{cc} X & 0\\ 0 & X \end{array}\right]$	$g_{5}$
$g_{6}$	$\left[\begin{array}{cc} 1 & 5\\ 8 & 9 \end{array}\right]$	$\left[\begin{array}{cc} 5 & 2\\ X & 6 \end{array}\right]$	$g_{6}$
$g_{7}$	$\left[\begin{array}{cc} 5 & 7\\ 8 & 5 \end{array}\right]$	$\left[\begin{array}{cc} 4 & 4\\ X & 7 \end{array}\right]$	$g_{7}$
$g_{8}$	$\left[\begin{array}{cc} 5 & 3\\ 2 & 5 \end{array}\right]$	$\left[\begin{array}{cc} 7 & 7\\ 1 & 4 \end{array}\right]$	$g_{8}$
$g_{9}$	$\left[\begin{array}{cc} 9 & 5\\ 2 & 1 \end{array}\right]$	$\left[\begin{array}{cc} 6 & 9\\ 1 & 5 \end{array}\right]$	$g_{9}$
$g_{10}$	$\left[\begin{array}{cc} 5 & 8\\ 8 & 9 \end{array}\right]$	$\left[\begin{array}{cc} 4 & X\\ X & 6 \end{array}\right]$	$g_{3}$
$g_{11}$	$\left[\begin{array}{cc} 1 & 8\\ 8 & 5 \end{array}\right]$	$\left[\begin{array}{cc} 5 & X\\ X & 7 \end{array}\right]$	$g_{4}$

These matrices are used to operate the elements of the group with the elements of the heading spaces, which in this case are $Z_{10}^{2}$ and $Z_{11}^{2}$. The elements of the heading spaces are denoted as column vectors and the operation by the elements of *G* is done using the standard multiplication of matrices. If we multiply $g_{i}$ with the head $\left[\begin{array}{c} u\\ v \end{array}\right]$, we first have to know where the head is. If it is in $Z_{10}^{2}$, we use the matrix associated to $g_{i}$ over $Z_{10}$ in Table A2, and, if the head is in $Z_{11}^{2}$, we use the matrix over $Z_{11}$ given in the same table.

We have two reduction maps that switch the representations between the two heading spaces. The reduction $R_{0}$, given in Table A3, takes values in $Z_{10}^{2}$ and gives the results in $Z_{11}^{2}$. The reduction $R_{1}$, given in Table A4, works the other way round. The values are given in Table A3 and  Table A4.

**Table A3:** Reduction 0.

$R_{0}$	0	1	2	3	4	5	6	7	8	9
0	$\left[\begin{array}{c} 4\\ 8 \end{array}\right]$	$\left[\begin{array}{c} 8\\ 0 \end{array}\right]$	$\left[\begin{array}{c} 8\\ 5 \end{array}\right]$	$\left[\begin{array}{c} 0\\ 3 \end{array}\right]$	$\left[\begin{array}{c} 1\\ 5 \end{array}\right]$	$\left[\begin{array}{c} 3\\ X \end{array}\right]$	$\left[\begin{array}{c} 1\\ 5 \end{array}\right]$	$\left[\begin{array}{c} 6\\ 4 \end{array}\right]$	$\left[\begin{array}{c} 7\\ 4 \end{array}\right]$	$\left[\begin{array}{c} 6\\ 9 \end{array}\right]$
1	$\left[\begin{array}{c} 1\\ X \end{array}\right]$	$\left[\begin{array}{c} 3\\ 0 \end{array}\right]$	$\left[\begin{array}{c} 0\\ 4 \end{array}\right]$	$\left[\begin{array}{c} 5\\ 8 \end{array}\right]$	$\left[\begin{array}{c} 3\\ 9 \end{array}\right]$	$\left[\begin{array}{c} 0\\ 2 \end{array}\right]$	$\left[\begin{array}{c} 2\\ 6 \end{array}\right]$	$\left[\begin{array}{c} 7\\ X \end{array}\right]$	$\left[\begin{array}{c} X\\ 6 \end{array}\right]$	$\left[\begin{array}{c} 7\\ 3 \end{array}\right]$
2	$\left[\begin{array}{c} 1\\ 5 \end{array}\right]$	$\left[\begin{array}{c} 0\\ X \end{array}\right]$	$\left[\begin{array}{c} 3\\ X \end{array}\right]$	$\left[\begin{array}{c} 0\\ 1 \end{array}\right]$	$\left[\begin{array}{c} 9\\ X \end{array}\right]$	$\left[\begin{array}{c} 9\\ 6 \end{array}\right]$	$\left[\begin{array}{c} 1\\ 5 \end{array}\right]$	$\left[\begin{array}{c} 8\\ 7 \end{array}\right]$	$\left[\begin{array}{c} 9\\ 5 \end{array}\right]$	$\left[\begin{array}{c} 2\\ 7 \end{array}\right]$
3	$\left[\begin{array}{c} 5\\ 1 \end{array}\right]$	$\left[\begin{array}{c} X\\ 5 \end{array}\right]$	$\left[\begin{array}{c} 5\\ 0 \end{array}\right]$	$\left[\begin{array}{c} 2\\ 5 \end{array}\right]$	$\left[\begin{array}{c} X\\ 8 \end{array}\right]$	$\left[\begin{array}{c} 3\\ 9 \end{array}\right]$	$\left[\begin{array}{c} 0\\ 6 \end{array}\right]$	$\left[\begin{array}{c} 5\\ 3 \end{array}\right]$	$\left[\begin{array}{c} 0\\ 1 \end{array}\right]$	$\left[\begin{array}{c} X\\ 2 \end{array}\right]$
4	$\left[\begin{array}{c} 4\\ 8 \end{array}\right]$	$\left[\begin{array}{c} 6\\ 7 \end{array}\right]$	$\left[\begin{array}{c} 7\\ 2 \end{array}\right]$	$\left[\begin{array}{c} 0\\ 8 \end{array}\right]$	$\left[\begin{array}{c} 7\\ 0 \end{array}\right]$	$\left[\begin{array}{c} 4\\ 7 \end{array}\right]$	$\left[\begin{array}{c} 4\\ 2 \end{array}\right]$	$\left[\begin{array}{c} 1\\ 7 \end{array}\right]$	$\left[\begin{array}{c} 3\\ 8 \end{array}\right]$	$\left[\begin{array}{c} 8\\ 8 \end{array}\right]$
5	$\left[\begin{array}{c} 1\\ 4 \end{array}\right]$	$\left[\begin{array}{c} X\\ 7 \end{array}\right]$	$\left[\begin{array}{c} 8\\ X \end{array}\right]$	$\left[\begin{array}{c} 2\\ 2 \end{array}\right]$	$\left[\begin{array}{c} 6\\ 3 \end{array}\right]$	$\left[\begin{array}{c} X\\ 6 \end{array}\right]$	$\left[\begin{array}{c} 6\\ 3 \end{array}\right]$	$\left[\begin{array}{c} 1\\ 8 \end{array}\right]$	$\left[\begin{array}{c} 9\\ 3 \end{array}\right]$	$\left[\begin{array}{c} 9\\ 6 \end{array}\right]$
6	$\left[\begin{array}{c} X\\ 3 \end{array}\right]$	$\left[\begin{array}{c} 3\\ 3 \end{array}\right]$	$\left[\begin{array}{c} X\\ 1 \end{array}\right]$	$\left[\begin{array}{c} 6\\ X \end{array}\right]$	$\left[\begin{array}{c} 9\\ 1 \end{array}\right]$	$\left[\begin{array}{c} 3\\ 4 \end{array}\right]$	$\left[\begin{array}{c} 6\\ 0 \end{array}\right]$	$\left[\begin{array}{c} 0\\ 8 \end{array}\right]$	$\left[\begin{array}{c} 2\\ 1 \end{array}\right]$	$\left[\begin{array}{c} 1\\ 4 \end{array}\right]$
7	$\left[\begin{array}{c} 5\\ 1 \end{array}\right]$	$\left[\begin{array}{c} 8\\ 0 \end{array}\right]$	$\left[\begin{array}{c} 3\\ 0 \end{array}\right]$	$\left[\begin{array}{c} 6\\ 8 \end{array}\right]$	$\left[\begin{array}{c} 3\\ X \end{array}\right]$	$\left[\begin{array}{c} 0\\ X \end{array}\right]$	$\left[\begin{array}{c} 1\\ 3 \end{array}\right]$	$\left[\begin{array}{c} 5\\ 4 \end{array}\right]$	$\left[\begin{array}{c} 5\\ 0 \end{array}\right]$	$\left[\begin{array}{c} 7\\ 9 \end{array}\right]$
8	$\left[\begin{array}{c} 1\\ 5 \end{array}\right]$	$\left[\begin{array}{c} 8\\ 1 \end{array}\right]$	$\left[\begin{array}{c} 2\\ 9 \end{array}\right]$	$\left[\begin{array}{c} 3\\ 6 \end{array}\right]$	$\left[\begin{array}{c} 1\\ 5 \end{array}\right]$	$\left[\begin{array}{c} X\\ 7 \end{array}\right]$	$\left[\begin{array}{c} 9\\ X \end{array}\right]$	$\left[\begin{array}{c} 0\\ 1 \end{array}\right]$	$\left[\begin{array}{c} 3\\ X \end{array}\right]$	$\left[\begin{array}{c} 7\\ 6 \end{array}\right]$
9	$\left[\begin{array}{c} 3\\ 1 \end{array}\right]$	$\left[\begin{array}{c} 9\\ 9 \end{array}\right]$	$\left[\begin{array}{c} 6\\ 2 \end{array}\right]$	$\left[\begin{array}{c} 0\\ 9 \end{array}\right]$	$\left[\begin{array}{c} 2\\ 6 \end{array}\right]$	$\left[\begin{array}{c} 0\\ 6 \end{array}\right]$	$\left[\begin{array}{c} 2\\ 9 \end{array}\right]$	$\left[\begin{array}{c} 2\\ 4 \end{array}\right]$	$\left[\begin{array}{c} 0\\ 7 \end{array}\right]$	$\left[\begin{array}{c} 3\\ 0 \end{array}\right]$

**Table A4:** Reduction 1.

$R_{1}$	0	1	2	3	4	5	6	7	8	9	X
0	$\left[\begin{array}{c} 7\\ 3 \end{array}\right]$	$\left[\begin{array}{c} 7\\ 3 \end{array}\right]$	$\left[\begin{array}{c} 1\\ 5 \end{array}\right]$	$\left[\begin{array}{c} 6\\ 9 \end{array}\right]$	$\left[\begin{array}{c} 8\\ 2 \end{array}\right]$	$\left[\begin{array}{c} 1\\ 7 \end{array}\right]$	$\left[\begin{array}{c} 9\\ 3 \end{array}\right]$	$\left[\begin{array}{c} 8\\ 2 \end{array}\right]$	$\left[\begin{array}{c} 4\\ 2 \end{array}\right]$	$\left[\begin{array}{c} 7\\ 6 \end{array}\right]$	$\left[\begin{array}{c} 2\\ 1 \end{array}\right]$
1	$\left[\begin{array}{c} 9\\ 7 \end{array}\right]$	$\left[\begin{array}{c} 4\\ 1 \end{array}\right]$	$\left[\begin{array}{c} 5\\ 7 \end{array}\right]$	$\left[\begin{array}{c} 9\\ 5 \end{array}\right]$	$\left[\begin{array}{c} 3\\ 3 \end{array}\right]$	$\left[\begin{array}{c} 4\\ 4 \end{array}\right]$	$\left[\begin{array}{c} 8\\ 3 \end{array}\right]$	$\left[\begin{array}{c} 3\\ 0 \end{array}\right]$	$\left[\begin{array}{c} 8\\ 1 \end{array}\right]$	$\left[\begin{array}{c} 5\\ 3 \end{array}\right]$	$\left[\begin{array}{c} 6\\ 6 \end{array}\right]$
2	$\left[\begin{array}{c} 9\\ 8 \end{array}\right]$	$\left[\begin{array}{c} 4\\ 1 \end{array}\right]$	$\left[\begin{array}{c} 8\\ 8 \end{array}\right]$	$\left[\begin{array}{c} 9\\ 2 \end{array}\right]$	$\left[\begin{array}{c} 4\\ 5 \end{array}\right]$	$\left[\begin{array}{c} 0\\ 3 \end{array}\right]$	$\left[\begin{array}{c} 9\\ 8 \end{array}\right]$	$\left[\begin{array}{c} 4\\ 0 \end{array}\right]$	$\left[\begin{array}{c} 8\\ 0 \end{array}\right]$	$\left[\begin{array}{c} 9\\ 4 \end{array}\right]$	$\left[\begin{array}{c} 0\\ 2 \end{array}\right]$
3	$\left[\begin{array}{c} 2\\ 9 \end{array}\right]$	$\left[\begin{array}{c} 7\\ 8 \end{array}\right]$	$\left[\begin{array}{c} 9\\ 3 \end{array}\right]$	$\left[\begin{array}{c} 4\\ 7 \end{array}\right]$	$\left[\begin{array}{c} 7\\ 9 \end{array}\right]$	$\left[\begin{array}{c} 2\\ 9 \end{array}\right]$	$\left[\begin{array}{c} 5\\ 4 \end{array}\right]$	$\left[\begin{array}{c} 8\\ 2 \end{array}\right]$	$\left[\begin{array}{c} 0\\ 4 \end{array}\right]$	$\left[\begin{array}{c} 2\\ 3 \end{array}\right]$	$\left[\begin{array}{c} 3\\ 9 \end{array}\right]$
4	$\left[\begin{array}{c} 4\\ 8 \end{array}\right]$	$\left[\begin{array}{c} 1\\ 5 \end{array}\right]$	$\left[\begin{array}{c} 9\\ 7 \end{array}\right]$	$\left[\begin{array}{c} 6\\ 3 \end{array}\right]$	$\left[\begin{array}{c} 5\\ 3 \end{array}\right]$	$\left[\begin{array}{c} 8\\ 7 \end{array}\right]$	$\left[\begin{array}{c} 3\\ 5 \end{array}\right]$	$\left[\begin{array}{c} 4\\ 6 \end{array}\right]$	$\left[\begin{array}{c} 1\\ 1 \end{array}\right]$	$\left[\begin{array}{c} 7\\ 7 \end{array}\right]$	$\left[\begin{array}{c} 1\\ 0 \end{array}\right]$
5	$\left[\begin{array}{c} 9\\ 2 \end{array}\right]$	$\left[\begin{array}{c} 4\\ 9 \end{array}\right]$	$\left[\begin{array}{c} 5\\ 3 \end{array}\right]$	$\left[\begin{array}{c} 8\\ 9 \end{array}\right]$	$\left[\begin{array}{c} 4\\ 3 \end{array}\right]$	$\left[\begin{array}{c} 7\\ 7 \end{array}\right]$	$\left[\begin{array}{c} 7\\ 7 \end{array}\right]$	$\left[\begin{array}{c} 8\\ 5 \end{array}\right]$	$\left[\begin{array}{c} 0\\ 8 \end{array}\right]$	$\left[\begin{array}{c} 9\\ 0 \end{array}\right]$	$\left[\begin{array}{c} 3\\ 3 \end{array}\right]$
6	$\left[\begin{array}{c} 5\\ 8 \end{array}\right]$	$\left[\begin{array}{c} 7\\ 9 \end{array}\right]$	$\left[\begin{array}{c} 9\\ 0 \end{array}\right]$	$\left[\begin{array}{c} 3\\ 1 \end{array}\right]$	$\left[\begin{array}{c} 5\\ 1 \end{array}\right]$	$\left[\begin{array}{c} 7\\ 7 \end{array}\right]$	$\left[\begin{array}{c} 8\\ 3 \end{array}\right]$	$\left[\begin{array}{c} 5\\ 9 \end{array}\right]$	$\left[\begin{array}{c} 3\\ 5 \end{array}\right]$	$\left[\begin{array}{c} 5\\ 3 \end{array}\right]$	$\left[\begin{array}{c} 6\\ 3 \end{array}\right]$
7	$\left[\begin{array}{c} 4\\ 8 \end{array}\right]$	$\left[\begin{array}{c} 5\\ 2 \end{array}\right]$	$\left[\begin{array}{c} 7\\ 7 \end{array}\right]$	$\left[\begin{array}{c} 5\\ 7 \end{array}\right]$	$\left[\begin{array}{c} 2\\ 7 \end{array}\right]$	$\left[\begin{array}{c} 9\\ 3 \end{array}\right]$	$\left[\begin{array}{c} 8\\ 7 \end{array}\right]$	$\left[\begin{array}{c} 5\\ 3 \end{array}\right]$	$\left[\begin{array}{c} 5\\ 3 \end{array}\right]$	$\left[\begin{array}{c} 0\\ 2 \end{array}\right]$	$\left[\begin{array}{c} 3\\ 4 \end{array}\right]$
8	$\left[\begin{array}{c} 9\\ 1 \end{array}\right]$	$\left[\begin{array}{c} 1\\ 9 \end{array}\right]$	$\left[\begin{array}{c} 8\\ 7 \end{array}\right]$	$\left[\begin{array}{c} 1\\ 0 \end{array}\right]$	$\left[\begin{array}{c} 8\\ 2 \end{array}\right]$	$\left[\begin{array}{c} 6\\ 5 \end{array}\right]$	$\left[\begin{array}{c} 2\\ 7 \end{array}\right]$	$\left[\begin{array}{c} 1\\ 3 \end{array}\right]$	$\left[\begin{array}{c} 4\\ 7 \end{array}\right]$	$\left[\begin{array}{c} 5\\ 1 \end{array}\right]$	$\left[\begin{array}{c} 6\\ 2 \end{array}\right]$
9	$\left[\begin{array}{c} 4\\ 6 \end{array}\right]$	$\left[\begin{array}{c} 9\\ 7 \end{array}\right]$	$\left[\begin{array}{c} 1\\ 6 \end{array}\right]$	$\left[\begin{array}{c} 1\\ 0 \end{array}\right]$	$\left[\begin{array}{c} 4\\ 0 \end{array}\right]$	$\left[\begin{array}{c} 2\\ 8 \end{array}\right]$	$\left[\begin{array}{c} 6\\ 8 \end{array}\right]$	$\left[\begin{array}{c} 4\\ 5 \end{array}\right]$	$\left[\begin{array}{c} 7\\ 3 \end{array}\right]$	$\left[\begin{array}{c} 6\\ 0 \end{array}\right]$	$\left[\begin{array}{c} 0\\ 7 \end{array}\right]$
X	$\left[\begin{array}{c} 6\\ 2 \end{array}\right]$	$\left[\begin{array}{c} 1\\ 8 \end{array}\right]$	$\left[\begin{array}{c} 5\\ 3 \end{array}\right]$	$\left[\begin{array}{c} 0\\ 1 \end{array}\right]$	$\left[\begin{array}{c} 7\\ 0 \end{array}\right]$	$\left[\begin{array}{c} 8\\ 3 \end{array}\right]$	$\left[\begin{array}{c} 5\\ 2 \end{array}\right]$	$\left[\begin{array}{c} 8\\ 6 \end{array}\right]$	$\left[\begin{array}{c} 9\\ 5 \end{array}\right]$	$\left[\begin{array}{c} 1\\ 1 \end{array}\right]$	$\left[\begin{array}{c} 1\\ 7 \end{array}\right]$

This algorithm requires an extra table to compute the nonlinear permutation *S*. This table is also used for the output and is called *B* (see Table A5).

**Table A5:** B map.

*B*	0	1	2	3	4	5	6	7	8	9
0	$\left[\begin{array}{c} 0\\ 0 \end{array}\right]$	$\left[\begin{array}{c} 7\\ 0 \end{array}\right]$	$\left[\begin{array}{c} 3\\ 1 \end{array}\right]$	$\left[\begin{array}{c} 0\\ 5 \end{array}\right]$	$\left[\begin{array}{c} 2\\ 3 \end{array}\right]$	$\left[\begin{array}{c} 0\\ 0 \end{array}\right]$	$\left[\begin{array}{c} 2\\ 3 \end{array}\right]$	$\left[\begin{array}{c} 2\\ 9 \end{array}\right]$	$\left[\begin{array}{c} 4\\ 3 \end{array}\right]$	$\left[\begin{array}{c} 0\\ 0 \end{array}\right]$
1	$\left[\begin{array}{c} 2\\ 2 \end{array}\right]$	$\left[\begin{array}{c} 8\\ 3 \end{array}\right]$	$\left[\begin{array}{c} 9\\ 1 \end{array}\right]$	$\left[\begin{array}{c} 1\\ 3 \end{array}\right]$	$\left[\begin{array}{c} 0\\ 0 \end{array}\right]$	$\left[\begin{array}{c} 3\\ 2 \end{array}\right]$	$\left[\begin{array}{c} 1\\ 4 \end{array}\right]$	$\left[\begin{array}{c} 0\\ 7 \end{array}\right]$	$\left[\begin{array}{c} 9\\ 5 \end{array}\right]$	$\left[\begin{array}{c} 9\\ 5 \end{array}\right]$
2	$\left[\begin{array}{c} 2\\ 3 \end{array}\right]$	$\left[\begin{array}{c} 1\\ 3 \end{array}\right]$	$\left[\begin{array}{c} 0\\ 2 \end{array}\right]$	$\left[\begin{array}{c} 1\\ 0 \end{array}\right]$	$\left[\begin{array}{c} 0\\ 2 \end{array}\right]$	$\left[\begin{array}{c} 8\\ 2 \end{array}\right]$	$\left[\begin{array}{c} 2\\ 3 \end{array}\right]$	$\left[\begin{array}{c} 3\\ 7 \end{array}\right]$	$\left[\begin{array}{c} 1\\ 8 \end{array}\right]$	$\left[\begin{array}{c} 6\\ 8 \end{array}\right]$
3	$\left[\begin{array}{c} 8\\ 2 \end{array}\right]$	$\left[\begin{array}{c} 3\\ 3 \end{array}\right]$	$\left[\begin{array}{c} 2\\ 1 \end{array}\right]$	$\left[\begin{array}{c} 6\\ 4 \end{array}\right]$	$\left[\begin{array}{c} 4\\ 0 \end{array}\right]$	$\left[\begin{array}{c} 6\\ 8 \end{array}\right]$	$\left[\begin{array}{c} 0\\ 0 \end{array}\right]$	$\left[\begin{array}{c} 6\\ 3 \end{array}\right]$	$\left[\begin{array}{c} 0\\ 0 \end{array}\right]$	$\left[\begin{array}{c} 0\\ 0 \end{array}\right]$
4	$\left[\begin{array}{c} 5\\ 3 \end{array}\right]$	$\left[\begin{array}{c} 0\\ 4 \end{array}\right]$	$\left[\begin{array}{c} 1\\ 1 \end{array}\right]$	$\left[\begin{array}{c} 3\\ 2 \end{array}\right]$	$\left[\begin{array}{c} 4\\ 3 \end{array}\right]$	$\left[\begin{array}{c} 1\\ 5 \end{array}\right]$	$\left[\begin{array}{c} 0\\ 8 \end{array}\right]$	$\left[\begin{array}{c} 4\\ 3 \end{array}\right]$	$\left[\begin{array}{c} 1\\ 9 \end{array}\right]$	$\left[\begin{array}{c} 6\\ 8 \end{array}\right]$
5	$\left[\begin{array}{c} 0\\ 0 \end{array}\right]$	$\left[\begin{array}{c} 7\\ 1 \end{array}\right]$	$\left[\begin{array}{c} 0\\ 3 \end{array}\right]$	$\left[\begin{array}{c} 1\\ 2 \end{array}\right]$	$\left[\begin{array}{c} 3\\ 2 \end{array}\right]$	$\left[\begin{array}{c} 0\\ 0 \end{array}\right]$	$\left[\begin{array}{c} 3\\ 2 \end{array}\right]$	$\left[\begin{array}{c} 9\\ 7 \end{array}\right]$	$\left[\begin{array}{c} 5\\ 7 \end{array}\right]$	$\left[\begin{array}{c} 8\\ 2 \end{array}\right]$
6	$\left[\begin{array}{c} 1\\ 8 \end{array}\right]$	$\left[\begin{array}{c} 6\\ 8 \end{array}\right]$	$\left[\begin{array}{c} 9\\ 5 \end{array}\right]$	$\left[\begin{array}{c} 0\\ 1 \end{array}\right]$	$\left[\begin{array}{c} 0\\ 8 \end{array}\right]$	$\left[\begin{array}{c} 3\\ 3 \end{array}\right]$	$\left[\begin{array}{c} 0\\ 9 \end{array}\right]$	$\left[\begin{array}{c} 3\\ 2 \end{array}\right]$	$\left[\begin{array}{c} 1\\ 0 \end{array}\right]$	$\left[\begin{array}{c} 6\\ 5 \end{array}\right]$
7	$\left[\begin{array}{c} 8\\ 2 \end{array}\right]$	$\left[\begin{array}{c} 7\\ 0 \end{array}\right]$	$\left[\begin{array}{c} 0\\ 0 \end{array}\right]$	$\left[\begin{array}{c} 6\\ 3 \end{array}\right]$	$\left[\begin{array}{c} 0\\ 0 \end{array}\right]$	$\left[\begin{array}{c} 1\\ 3 \end{array}\right]$	$\left[\begin{array}{c} 4\\ 0 \end{array}\right]$	$\left[\begin{array}{c} 3\\ 3 \end{array}\right]$	$\left[\begin{array}{c} 2\\ 1 \end{array}\right]$	$\left[\begin{array}{c} 4\\ 9 \end{array}\right]$
8	$\left[\begin{array}{c} 2\\ 3 \end{array}\right]$	$\left[\begin{array}{c} 0\\ 8 \end{array}\right]$	$\left[\begin{array}{c} 1\\ 8 \end{array}\right]$	$\left[\begin{array}{c} 4\\ 5 \end{array}\right]$	$\left[\begin{array}{c} 2\\ 3 \end{array}\right]$	$\left[\begin{array}{c} 7\\ 1 \end{array}\right]$	$\left[\begin{array}{c} 0\\ 2 \end{array}\right]$	$\left[\begin{array}{c} 1\\ 0 \end{array}\right]$	$\left[\begin{array}{c} 0\\ 2 \end{array}\right]$	$\left[\begin{array}{c} 6\\ 8 \end{array}\right]$
9	$\left[\begin{array}{c} 1\\ 0 \end{array}\right]$	$\left[\begin{array}{c} 0\\ 9 \end{array}\right]$	$\left[\begin{array}{c} 0\\ 6 \end{array}\right]$	$\left[\begin{array}{c} 0\\ 7 \end{array}\right]$	$\left[\begin{array}{c} 1\\ 4 \end{array}\right]$	$\left[\begin{array}{c} 8\\ 2 \end{array}\right]$	$\left[\begin{array}{c} 0\\ 0 \end{array}\right]$	$\left[\begin{array}{c} 1\\ 0 \end{array}\right]$	$\left[\begin{array}{c} 9\\ 1 \end{array}\right]$	$\left[\begin{array}{c} 8\\ 3 \end{array}\right]$

For each possible input, we need the values that are used to compute it in the algorithm. The  first input ($v_{0}$) is introduced using a head in $Z_{10}^{2}$ and the other input values is introduced using three group values for each input. The values are given in Table A6.

**Table A6:** Input table.

IT	$v_{0}$	$v_{1}$			$v_{2}$			$v_{3}$			$v_{4}$		
*i*	0	1	2	3	4	5	6	7	8	9	10	11	12
0	$\left[\begin{array}{c} 3\\ 3 \end{array}\right]$	$g_{1}$	$g_{8}$	$g_{9}$	$g_{6}$	$g_{1}$	$g_{6}$	$g_{4}$	$g_{3}$	$g_{1}$	$g_{11}$	$g_{6}$	$g_{3}$
1	$\left[\begin{array}{c} 8\\ 2 \end{array}\right]$	$g_{8}$	$g_{5}$	$g_{11}$	$g_{8}$	$g_{7}$	$g_{4}$	$g_{9}$	$g_{4}$	$g_{3}$	$g_{1}$	$g_{1}$	$g_{4}$
2	$\left[\begin{array}{c} 1\\ 8 \end{array}\right]$	$g_{6}$	$g_{7}$	$g_{0}$	$g_{7}$	$g_{10}$	$g_{1}$	$g_{1}$	$g_{4}$	$g_{11}$	$g_{11}$	$g_{1}$	$g_{2}$
3	$\left[\begin{array}{c} 0\\ 1 \end{array}\right]$	$g_{3}$	$g_{5}$	$g_{3}$	$g_{2}$	$g_{2}$	$g_{5}$	$g_{9}$	$g_{11}$	$g_{4}$	$g_{11}$	$g_{6}$	$g_{8}$
4	$\left[\begin{array}{c} 6\\ 8 \end{array}\right]$	$g_{9}$	$g_{8}$	$g_{9}$	$g_{6}$	$g_{8}$	$g_{4}$	$g_{11}$	$g_{8}$	$g_{7}$	$g_{9}$	$g_{10}$	$g_{8}$
5	$\left[\begin{array}{c} 9\\ 5 \end{array}\right]$	$g_{11}$	$g_{1}$	$g_{5}$	$g_{1}$	$g_{4}$	$g_{7}$	$g_{0}$	$g_{3}$	$g_{3}$	$g_{0}$	$g_{9}$	$g_{9}$
6	$\left[\begin{array}{c} 1\\ 0 \end{array}\right]$	$g_{2}$	$g_{10}$	$g_{0}$	$g_{6}$	$g_{3}$	$g_{7}$	$g_{1}$	$g_{6}$	$g_{5}$	$g_{8}$	$g_{3}$	$g_{7}$
7	$\left[\begin{array}{c} 6\\ 7 \end{array}\right]$	$g_{3}$	$g_{1}$	$g_{8}$	$g_{4}$	$g_{2}$	$g_{1}$	$g_{1}$	$g_{3}$	$g_{0}$	$g_{1}$	$g_{4}$	$g_{1}$
8	$\left[\begin{array}{c} 3\\ 1 \end{array}\right]$	$g_{3}$	$g_{8}$	$g_{1}$	$g_{6}$	$g_{0}$	$g_{1}$	$g_{0}$	$g_{11}$	$g_{5}$	$g_{6}$	$g_{9}$	$g_{11}$
9	$\left[\begin{array}{c} 7\\ 8 \end{array}\right]$	$g_{5}$	$g_{11}$	$g_{5}$	$g_{4}$	$g_{1}$	$g_{7}$	$g_{3}$	$g_{9}$	$g_{7}$	$g_{1}$	$g_{7}$	$g_{5}$
a	$\left[\begin{array}{c} 0\\ 2 \end{array}\right]$	$g_{10}$	$g_{7}$	$g_{0}$	$g_{6}$	$g_{0}$	$g_{5}$	$g_{11}$	$g_{6}$	$g_{8}$	$g_{5}$	$g_{4}$	$g_{5}$
b	$\left[\begin{array}{c} 1\\ 9 \end{array}\right]$	$g_{2}$	$g_{6}$	$g_{2}$	$g_{5}$	$g_{6}$	$g_{0}$	$g_{5}$	$g_{8}$	$g_{2}$	$g_{1}$	$g_{8}$	$g_{10}$
c	$\left[\begin{array}{c} 7\\ 0 \end{array}\right]$	$g_{8}$	$g_{5}$	$g_{9}$	$g_{4}$	$g_{6}$	$g_{1}$	$g_{3}$	$g_{10}$	$g_{1}$	$g_{1}$	$g_{6}$	$g_{11}$
d	$\left[\begin{array}{c} 1\\ 3 \end{array}\right]$	$g_{1}$	$g_{8}$	$g_{11}$	$g_{5}$	$g_{0}$	$g_{2}$	$g_{2}$	$g_{8}$	$g_{3}$	$g_{8}$	$g_{5}$	$g_{0}$
e	$\left[\begin{array}{c} 2\\ 3 \end{array}\right]$	$g_{10}$	$g_{7}$	$g_{6}$	$g_{4}$	$g_{8}$	$g_{2}$	$g_{8}$	$g_{10}$	$g_{9}$	$g_{8}$	$g_{9}$	$g_{7}$
f	$\left[\begin{array}{c} 2\\ 9 \end{array}\right]$	$g_{8}$	$g_{11}$	$g_{8}$	$g_{10}$	$g_{9}$	$g_{4}$	$g_{3}$	$g_{1}$	$g_{6}$	$g_{9}$	$g_{8}$	$g_{10}$

The reduction step in this algorithm, which is called step, swaps the heads between $Z_{10}^{2}$ and $Z_{11}^{2}$.

**Algorithm A2** Basic reduction step.
Require: *w* is a head, $g_{i}\in G$ 1: **if** $w\in Z_{10}^{2}$ **then** 2: **return** $g_{i}R_{0}\left(g_{i}^{-1}w\right)$ 3: **else** {$w\in Z_{11}^{2}$} 4: **return** $g_{i}R_{1}\left(g_{i}^{-1}w\right)$ 5: **end if**

The multiplication of the elements of a group by the heads is done with the matrix representation of the group elements. Therefore, in the computation $g_{i}R_{j}\left(g_{i}^{-1}w\right)$, the matrices corresponding to $g_{i}$ and $g_{i}^{-1}$ are over different rings. The full implementation is given in Algorithm A3.

The last public element of the implementation is the program itself.

**Algorithm A3** Public program (obfuscated version).
1: $w\leftarrow IT_{0}\left(v_{0}\right)$ 2: $w\leftarrow \mathrm{step}(IT_{1}\left(v_{1}\right),w)$ 3: $w\leftarrow \mathrm{step}(g_{6},w)$ 4: $w\leftarrow \mathrm{step}(IT_{2}\left(v_{1}\right),w)$ 5: $w\leftarrow \mathrm{step}(IT_{3}\left(v_{1}\right),w)$ 6: w←B(w) 7: $w\leftarrow \mathrm{step}(IT_{4}\left(v_{2}\right),w)$ 8: $w\leftarrow \mathrm{step}(g_{4},w)$ 9: $w\leftarrow \mathrm{step}(IT_{5}\left(v_{2}\right),w)$ 10: $w\leftarrow \mathrm{step}(IT_{6}\left(v_{2}\right),w)$ 11: w←B(w) 12: $w\leftarrow \mathrm{step}(IT_{7}\left(v_{3}\right),w)$ 13: $w\leftarrow \mathrm{step}(g_{10},w)$ 14: $w\leftarrow \mathrm{step}(IT_{8}\left(v_{3}\right),w)$ 15: $w\leftarrow \mathrm{step}(IT_{9}\left(v_{3}\right),w)$ 16: w←B(w) 17: $w\leftarrow \mathrm{step}(IT_{10}\left(v_{4}\right),w)$ 18: $w\leftarrow \mathrm{step}(g_{11},w)$ 19: $w\leftarrow \mathrm{step}(IT_{11}\left(v_{4}\right),w)$ 20: $w\leftarrow \mathrm{step}(IT_{12}\left(v_{4}\right),w)$ 21: w←B(w) 22: **return** numeric value of *w*

We see an execution of the whole function with the input values $v_{0}=1$,$v_{1}=6$,$v_{2}=a$,$v_{3}=5$ and $v_{4}=b$.

1: $w\leftarrow \left[\begin{array}{c} 8\\ 2 \end{array}\right]$
2: $w\leftarrow g_{2}R_{0}\left(g_{2}^{-1}w\right)=\left[\begin{array}{cc} 4 & 4\\ X & 7 \end{array}\right]R_{0}\left(\left[\begin{array}{cc} 5 & 7\\ 8 & 5 \end{array}\right]\left[\begin{array}{c} 8\\ 2 \end{array}\right]\right)=\left[\begin{array}{c} 6\\ 4 \end{array}\right]$
3: $w\leftarrow g_{6}R_{1}\left(g_{6}^{-1}w\right)=\left[\begin{array}{cc} 9 & 5\\ 2 & 1 \end{array}\right]R_{1}\left(\left[\begin{array}{cc} 6 & 9\\ 1 & 5 \end{array}\right]\left[\begin{array}{c} 6\\ 4 \end{array}\right]\right)=\left[\begin{array}{c} 0\\ 1 \end{array}\right]$
4: $w\leftarrow g_{10}R_{0}\left(g_{10}^{-1}w\right)=\left[\begin{array}{cc} 4 & X\\ X & 6 \end{array}\right]R_{0}\left(\left[\begin{array}{cc} 9 & 2\\ 2 & 5 \end{array}\right]\left[\begin{array}{c} 0\\ 1 \end{array}\right]\right)=\left[\begin{array}{c} 8\\ 5 \end{array}\right]$
5: $w\leftarrow g_{0}R_{1}\left(g_{0}^{-1}w\right)=\left[\begin{array}{cc} 1 & 0\\ 0 & 1 \end{array}\right]R_{1}\left(\left[\begin{array}{cc} 1 & 0\\ 0 & 1 \end{array}\right]\left[\begin{array}{c} 8\\ 5 \end{array}\right]\right)=\left[\begin{array}{c} 6\\ 5 \end{array}\right]$
6: $w\leftarrow B\left(\left[\begin{array}{c} 6\\ 5 \end{array}\right]\right)=\left[\begin{array}{c} 3\\ 3 \end{array}\right]$
7: $w\leftarrow g_{6}R_{0}\left(g_{6}^{-1}w\right)=\left[\begin{array}{cc} 6 & 9\\ 1 & 5 \end{array}\right]R_{0}\left(\left[\begin{array}{cc} 9 & 5\\ 2 & 1 \end{array}\right]\left[\begin{array}{c} 3\\ 3 \end{array}\right]\right)=\left[\begin{array}{c} 9\\ 4 \end{array}\right]$
8: $w\leftarrow g_{4}R_{1}\left(g_{4}^{-1}w\right)=\left[\begin{array}{cc} 9 & 2\\ 2 & 5 \end{array}\right]R_{1}\left(\left[\begin{array}{cc} 4 & X\\ X & 6 \end{array}\right]\left[\begin{array}{c} 9\\ 4 \end{array}\right]\right)=\left[\begin{array}{c} 3\\ 4 \end{array}\right]$
9: $w\leftarrow g_{0}R_{0}\left(g_{0}^{-1}w\right)=\left[\begin{array}{cc} 1 & 0\\ 0 & 1 \end{array}\right]R_{0}\left(\left[\begin{array}{cc} 1 & 0\\ 0 & 1 \end{array}\right]\left[\begin{array}{c} 3\\ 4 \end{array}\right]\right)=\left[\begin{array}{c} X\\ 8 \end{array}\right]$
10: $w\leftarrow g_{5}R_{1}\left(g_{5}^{-1}w\right)=\left[\begin{array}{cc} 5 & 3\\ 2 & 5 \end{array}\right]R_{1}\left(\left[\begin{array}{cc} 7 & 7\\ 1 & 4 \end{array}\right]\left[\begin{array}{c} X\\ 8 \end{array}\right]\right)=\left[\begin{array}{c} 5\\ 8 \end{array}\right]$
11: $w\leftarrow B\left(\left[\begin{array}{c} 5\\ 8 \end{array}\right]\right)=\left[\begin{array}{c} 5\\ 7 \end{array}\right]$
12: $w\leftarrow g_{0}R_{0}\left(g_{0}^{-1}w\right)=\left[\begin{array}{cc} 1 & 0\\ 0 & 1 \end{array}\right]R_{0}\left(\left[\begin{array}{cc} 1 & 0\\ 0 & 1 \end{array}\right]\left[\begin{array}{c} 5\\ 7 \end{array}\right]\right)=\left[\begin{array}{c} 1\\ 8 \end{array}\right]$
13: $w\leftarrow g_{10}R_{1}\left(g_{10}^{-1}w\right)=\left[\begin{array}{cc} 5 & 8\\ 8 & 9 \end{array}\right]R_{1}\left(\left[\begin{array}{cc} 6 & 1\\ 1 & 4 \end{array}\right]\left[\begin{array}{c} 1\\ 8 \end{array}\right]\right)=\left[\begin{array}{c} 2\\ 7 \end{array}\right]$
14: $w\leftarrow g_{3}R_{0}\left(g_{3}^{-1}w\right)=\left[\begin{array}{cc} 7 & 1\\ 1 & 5 \end{array}\right]R_{0}\left(\left[\begin{array}{cc} 1 & 8\\ 8 & 5 \end{array}\right]\left[\begin{array}{c} 2\\ 7 \end{array}\right]\right)=\left[\begin{array}{c} 2\\ 2 \end{array}\right]$
15: $w\leftarrow g_{3}R_{1}\left(g_{3}^{-1}w\right)=\left[\begin{array}{cc} 5 & 2\\ 2 & 1 \end{array}\right]R_{1}\left(\left[\begin{array}{cc} 5 & X\\ X & 7 \end{array}\right]\left[\begin{array}{c} 2\\ 2 \end{array}\right]\right)=\left[\begin{array}{c} 3\\ 1 \end{array}\right]$
16: $w\leftarrow B\left(\left[\begin{array}{c} 3\\ 1 \end{array}\right]\right)=\left[\begin{array}{c} 3\\ 3 \end{array}\right]$
17: $w\leftarrow g_{1}R_{0}\left(g_{1}^{-1}w\right)=\left[\begin{array}{cc} X & 2\\ 0 & 1 \end{array}\right]R_{0}\left(\left[\begin{array}{cc} 9 & 7\\ 0 & 1 \end{array}\right]\left[\begin{array}{c} 3\\ 3 \end{array}\right]\right)=\left[\begin{array}{c} 9\\ 6 \end{array}\right]$
18: $w\leftarrow g_{11}R_{1}\left(g_{11}^{-1}w\right)=\left[\begin{array}{cc} 9 & 0\\ 0 & 9 \end{array}\right]R_{1}\left(\left[\begin{array}{cc} X & 0\\ 0 & X \end{array}\right]\left[\begin{array}{c} 9\\ 6 \end{array}\right]\right)=\left[\begin{array}{c} 0\\ 7 \end{array}\right]$
19: $w\leftarrow g_{8}R_{0}\left(g_{8}^{-1}w\right)=\left[\begin{array}{cc} 5 & 2\\ X & 6 \end{array}\right]R_{0}\left(\left[\begin{array}{cc} 1 & 5\\ 8 & 9 \end{array}\right]\left[\begin{array}{c} 0\\ 7 \end{array}\right]\right)=\left[\begin{array}{c} 3\\ X \end{array}\right]$
20: $w\leftarrow g_{10}R_{1}\left(g_{10}^{-1}w\right)=\left[\begin{array}{cc} 5 & 8\\ 8 & 9 \end{array}\right]R_{1}\left(\left[\begin{array}{cc} 6 & 1\\ 1 & 4 \end{array}\right]\left[\begin{array}{c} 3\\ X \end{array}\right]\right)=\left[\begin{array}{c} 4\\ 5 \end{array}\right]$
21: $w\leftarrow B\left(\left[\begin{array}{c} 4\\ 5 \end{array}\right]\right)=\left[\begin{array}{c} 1\\ 5 \end{array}\right]$
22: **return** 10·1+5=f

### Appendix B.3. Obfuscated Version (Private Elements)

Beyond this point, we look at the information that is not available to the attacker, whose only visible elements are those given in the previous subsection. Some of the elements might be guessed by the attacker, but they are not visible.

The keys used in this implementation are $k_{0}=c$, $k_{1}=8$, $k_{2}=1$, $k_{3}=f$, $k_{4}=1$ and $k_{5}=4$. We can check that the obfuscated computation of ξ(1,6,a,5,b) given in the previous subsection is correct using Algorithm A1 and the keys provided.

1: $w_{1}\leftarrow S\left(k_{0}⊕v_{0}\right)=S\left(d\right)=9$
2: $w_{2}\leftarrow S\left(k_{1}⊕v_{1}⊕w_{1}\right)=S\left(7\right)=8$
3: $w_{3}\leftarrow S\left(k_{2}⊕v_{2}⊕w_{2}\right)=S\left(3\right)=1$
4: $w_{4}\leftarrow S\left(k_{3}⊕v_{3}⊕w_{3}\right)=S\left(b\right)=c$
5: $w_{5}\leftarrow S\left(k_{4}⊕v_{4}⊕w_{4}\right)=S\left(6\right)=b$
6: **return** $k_{5}⊕w_{5}=f$

One of the things that could be surprising at first sight to someone not used to this arithmetic is that, although we make computations in the vector space $V=F_{2}^{4}$, we use matrices over the rings $Z_{10}$ and $Z_{11}$. We only need heading spaces that let us define the actions of the group *G* up to isomorphism, and in this case the group can be described using these particular matrices.

We give an alternative presentation of the public group *G* that is used to define further private elements of the obfuscation. The group *G* can be described as a non-commutative group generated by three elements, u,v and *w*, such that $u^{3}=v^{2}=w^{2}=1$. The element *w* commutes with all the others, but the other two do not commute because $uv=vu^{2}$. All the elements of the group can be written as $w^{i}v^{j}v^{k}$ for i∈{0,1}, j∈{0,1} and k∈{0,1,2}. One possible correspondence between the elements of *G* that we have been using and this representation is given in Table A7.

**Table A7:** Group correspondences.

Group	$w^{i}v^{j}u^{k}$
$g_{0}$	1
$g_{1}$	wvu
$g_{2}$	vu
$g_{3}$	$u^{2}$
$g_{4}$	wu
$g_{5}$	*w*
$g_{6}$	$wvu^{2}$
$g_{7}$	*v*
$g_{8}$	wv
$g_{9}$	$vu^{2}$
$g_{10}$	*u*
$g_{11}$	$wu^{2}$

Although this group can be found using 4×4-matrices over $F_{2}$ (in fact, there are 30,240 ways to select u,v and *w* matching the conditions in ${\mathrm{Mat}}_{4\times 4}\left(F_{2}\right)$), the implementation uses the internal commutative group $M=F_{2}^{6}$ and thus 6×6 matrices over $F_{2}$ to represent the endomorphisms.

The computation of the real value represented by each element and the control values is done with the matrix $P=\left[\begin{array}{cccccc} 1 & 1 & 1 & 0 & 0 & 1\\ 1 & 1 & 0 & 1 & 0 & 0\\ 0 & 1 & 1 & 1 & 0 & 0\\ 0 & 1 & 0 & 0 & 1 & 1\\ 1 & 1 & 1 & 0 & 1 & 0\\ 1 & 1 & 1 & 1 & 0 & 1 \end{array}\right]$.

The first four bits are the projection and the last two are a control value. In this case, the control value is computed in the same way during the program.

The representation of *G* using matrices in ${\mathrm{Mat}}_{6\times 6}\left(F_{2}\right)$ is made using the matrices

$$u=\left[\begin{array}{cccccc} 1 & 0 & 1 & 1 & 1 & 0\\ 1 & 0 & 1 & 1 & 1 & 1\\ 1 & 0 & 0 & 0 & 1 & 1\\ 1 & 1 & 0 & 1 & 1 & 1\\ 1 & 1 & 0 & 1 & 0 & 0\\ 1 & 1 & 0 & 0 & 0 & 1 \end{array}\right]\;\;,v=\left[\begin{array}{cccccc} 1 & 0 & 1 & 0 & 0 & 0\\ 0 & 0 & 0 & 1 & 0 & 0\\ 0 & 0 & 1 & 0 & 0 & 0\\ 0 & 1 & 0 & 0 & 0 & 0\\ 0 & 1 & 0 & 1 & 0 & 1\\ 0 & 1 & 0 & 1 & 1 & 0 \end{array}\right]\mathrm{and}\;w=\left[\begin{array}{cccccc} 1 & 1 & 1 & 1 & 1 & 1\\ 1 & 0 & 1 & 1 & 1 & 0\\ 0 & 0 & 1 & 0 & 0 & 0\\ 1 & 0 & 0 & 1 & 0 & 1\\ 0 & 0 & 0 & 0 & 1 & 0\\ 0 & 1 & 1 & 1 & 1 & 0 \end{array}\right]\;\;.$$

The action of the group *G* on $M=F_{2}^{6}$ using these matrices generates 11 orbits and all of them are valid elements in this implementation. For each of them, we fix an element $m_{i}$ that is the base point to generate the orbit. The other elements of the orbit are $Gm_{i}=\left\{gm_{i}:g\in G\right\}$. For each of these representatives, we have to compute the stabilizer to find other elements in the heading spaces that could represent them. The list of representatives and stabilizers over the vector space $F_{2}^{6}$ is given in Table A8. The vectors in $F_{2}^{6}$ are represented in horizontal form, but they should be considered column vectors.

**Table A8:** Orbits representatives and stabilizers.

Representative	Stabilizer
$m_{0}=\left(0,0,0,0,0,0\right)$	$G_{m_{0}}=G$
$m_{1}=\left(1,1,1,0,1,0\right)$	$G_{m_{1}}=\left\{1,vu^{2}\right\}$
$m_{2}=\left(1,0,0,0,1,0\right)$	$G_{m_{2}}=\left\{1,vu^{2}\right\}$
$m_{3}=\left(1,0,1,0,0,0\right)$	$G_{m_{3}}=\left\{1\right\}$
$m_{4}=\left(0,1,1,0,0,0\right)$	$G_{m_{4}}=\left\{1,vu^{2},w,wvu^{2}\right\}$
$m_{5}=\left(0,0,0,1,0,0\right)$	$G_{m_{5}}=\left\{1,wvu\right\}$
$m_{6}=\left(1,0,0,1,0,0\right)$	$G_{m_{6}}=\left\{1\right\}$
$m_{7}=\left(1,1,0,0,0,1\right)$	$G_{m_{7}}=\left\{1,vu,w,wvu\right\}$
$m_{8}=\left(1,0,1,0,0,1\right)$	$G_{m_{8}}=\left\{1,w\right\}$
$m_{9}=\left(0,1,1,0,1,0\right)$	$G_{m_{9}}=\left\{1,wvu\right\}$
$m_{10}=\left(1,1,1,1,0,1\right)$	$G_{m_{10}}=\left\{1,vu^{2},w,wvu^{2}\right\}$

If we use the action over the heading spaces with the corresponding matrices given in Table A2, we get more than 11 orbits, but we only need to consider representatives that can extend the arithmetic of the $m_{i}$. Strictly speaking, the heading spaces are the subsets of $Z_{10}^{2}$ and $Z_{11}^{2}$ that belong to the orbits that we select for the representation, but this is not a problem because we define the tables over the other elements with some random values, and they are not reached during a legitimate execution of the program (unless we introduce fake instructions).

As we have seen, the construction of the correspondences between the heads and the elements of *M* requires the selection of elements $x_{i}$ and $y_{i}$ in the heading spaces such that that the stabilizer of the element(s) that represents $m_{i}$ in the heading spaces is (are) contained in the stabilizer of $m_{i}$. In this case, we only use one representative for each $m_{i}$, although more can be used. This would increase the number of possible representations for each element. In this case, we have multiple representations because, in some cases, the stabilizers of the elements in the heading spaces are strictly smaller than the stabilizers of the corresponding values $m_{i}$. The tables with the corresponding representative in the heading spaces and the stabilizers of these representatives are given in Table A9 for the heading space $Z_{10}^{2}$ and Table A10 for the heading space $Z_{11}^{2}$.

**Table A9:** Representatives in heading space $Z_{10}^{2}$.

Representative	Stabilizer
$x_{0}=\left[\begin{array}{c} 8\\ 0 \end{array}\right]$	$G_{x_{0}}=\left\{1,vu\right\}\subseteq G=G_{m_{0}}$
$x_{1}=\left[\begin{array}{c} 0\\ 8 \end{array}\right]$	$G_{x_{1}}=\left\{1,vu^{2}\right\}\subseteq \left\{1,vu^{2}\right\}=G_{m_{1}}$
$x_{2}=\left[\begin{array}{c} 5\\ 2 \end{array}\right]$	$G_{x_{2}}=\left\{1,vu^{2}\right\}\subseteq \left\{1,vu^{2}\right\}=G_{m_{2}}$
$x_{3}=\left[\begin{array}{c} 4\\ 7 \end{array}\right]$	$G_{x_{3}}=\left\{1\right\}\subseteq \left\{1\right\}=G_{m_{3}}$
$x_{4}=\left[\begin{array}{c} 5\\ 6 \end{array}\right]$	$G_{x_{4}}=\left\{1,vu^{2}\right\}\subseteq \left\{1,vu^{2},w,wvu^{2}\right\}=G_{m_{4}}$
$x_{5}=\left[\begin{array}{c} 6\\ 6 \end{array}\right]$	$G_{x_{5}}=\left\{1,wvu\right\}\subseteq \left\{1,wvu\right\}=G_{m_{5}}$
$x_{6}=\left[\begin{array}{c} 6\\ 9 \end{array}\right]$	$G_{x_{6}}=\left\{1\right\}\subseteq \left\{1\right\}=G_{m_{6}}$
$x_{7}=\left[\begin{array}{c} 1\\ 6 \end{array}\right]$	$G_{x_{7}}=\left\{1,wvu\right\}\subseteq \left\{1,vu,w,wvu\right\}=G_{m_{7}}$
$x_{8}=\left[\begin{array}{c} 3\\ 7 \end{array}\right]$	$G_{x_{8}}=\left\{1\right\}\subseteq \left\{1,w\right\}=G_{m_{8}}$
$x_{9}=\left[\begin{array}{c} 7\\ 1 \end{array}\right]$	$G_{x_{9}}=\left\{1\right\}\subseteq \left\{1,wvu\right\}=G_{m_{9}}$
$x_{10}=\left[\begin{array}{c} 6\\ 4 \end{array}\right]$	$G_{x_{10}}=\left\{1,wvu^{2}\right\}\subseteq \left\{1,vu^{2},w,wvu^{2}\right\}=G_{m_{10}}$

**Table A10:** Representatives in heading space $Z_{11}^{2}$.

Representative	Stabilizer
$y_{0}=\left[\begin{array}{c} 6\\ 9 \end{array}\right]$	$G_{y_{0}}=\left\{1,wv\right\}\subseteq G=G_{m_{0}}$
$y_{1}=\left[\begin{array}{c} 2\\ 5 \end{array}\right]$	$G_{y_{1}}=\left\{1,vu^{2}\right\}\subseteq \left\{1,vu^{2}\right\}=G_{m_{1}}$
$y_{2}=\left[\begin{array}{c} 1\\ 8 \end{array}\right]$	$G_{y_{2}}=\left\{1,vu^{2}\right\}\subseteq \left\{1,vu^{2}\right\}=G_{m_{2}}$
$y_{3}=\left[\begin{array}{c} 2\\ 6 \end{array}\right]$	$G_{y_{3}}=\left\{1\right\}\subseteq \left\{1\right\}=G_{m_{3}}$
$y_{4}=\left[\begin{array}{c} 6\\ 5 \end{array}\right]$	$G_{y_{4}}=\left\{1\right\}\subseteq \left\{1,vu^{2},w,wvu^{2}\right\}=G_{m_{4}}$
$y_{5}=\left[\begin{array}{c} 9\\ 9 \end{array}\right]$	$G_{y_{5}}=\left\{1,wvu\right\}\subseteq \left\{1,wvu\right\}=G_{m_{5}}$
$y_{6}=\left[\begin{array}{c} 8\\ 7 \end{array}\right]$	$G_{y_{6}}=\left\{1\right\}\subseteq \left\{1\right\}=G_{m_{6}}$
$y_{7}=\left[\begin{array}{c} 3\\ 3 \end{array}\right]$	$G_{y_{7}}=\left\{1,wvu\right\}\subseteq \left\{1,vu,w,wvu\right\}=G_{m_{7}}$
$y_{8}=\left[\begin{array}{c} 0\\ 6 \end{array}\right]$	$G_{y_{8}}=\left\{1\right\}\subseteq \left\{1,w\right\}=G_{m_{8}}$
$y_{9}=\left[\begin{array}{c} 8\\ 3 \end{array}\right]$	$G_{y_{9}}=\left\{1\right\}\subseteq \left\{1,wvu\right\}=G_{m_{9}}$
$y_{10}=\left[\begin{array}{c} 5\\ 7 \end{array}\right]$	$G_{y_{10}}=\left\{1,vu^{2}\right\}\subseteq \left\{1,vu^{2},w,wvu^{2}\right\}=G_{m_{10}}$

To build the internal group of the obfuscation, we consider the group *H*, which is a cyclic group of order 3 generated by an element *h*. This group has a nontrivial automorphism τ:H→H given by $\tau \left(h^{t}\right)=h^{-t}$ and we can use it to define φ:G→Aut(H) by $\varphi \left(w^{i}v^{j}u^{k}\right)=\tau^{i}$. This is a group homomorphism because the subgroup generated by *w* is a direct summand of *G*. This direct summand has order 2 and thus it is isomorphic to Aut(H). The homomorphism φ can be considered as the projection over the subgroup generated by *w* and the composition with the isomorphism between this subgroup and Aut(H).

Using φ, we can generate the semi-direct product $H{⋊}_{\varphi }G$. This is the internal group of the obfuscation and it has 36 elements. This group can be represented on Aut(M) using the matrix $h=\left[\begin{array}{cccccc} 1 & 1 & 0 & 1 & 0 & 0\\ 1 & 0 & 1 & 1 & 1 & 0\\ 0 & 0 & 1 & 0 & 0 & 0\\ 1 & 0 & 0 & 1 & 0 & 1\\ 1 & 0 & 1 & 1 & 1 & 1\\ 1 & 1 & 1 & 0 & 0 & 0 \end{array}\right]$ and the previous definitions of u,v and *w*. This means $H{⋊}_{\varphi }G$ is a subgroup of Aut(M). Note that $H{⋊}_{\varphi }G$ cannot be given by an extension of *G* in ${\mathrm{Mat}}_{2\times 2}\left(Z_{11}\right)$, but this is not necessary because we need actions only for the group *G* over the heading spaces.

We have only one base point b=(1,0,1,0,1,1). Using these values, we can compute the values associated to the heads and links. Suppose *g* is a link with *H*-modifier $h^{2}$, then the real value is $h^{2}gb\in M$ and we can use the matrix *P* to compute $Ph^{2}gb$, whose first four bits would be the value in *V* and the last two the control value.

Given a head $x\in Z_{10}^{2}$ with *H*-modifier *h*, we have to search for a group value *g* and a representative of the orbit $x_{i}$ such that $x=gx_{i}$. This value *g* is a 2×2 matrix over $Z_{10}$, but we have to get the matrix associated to the same group element, but in ${\mathrm{Mat}}_{6\times 6}\left(F_{2}\right)$, to compute $gm_{i}$ and finally apply the *H*-modifier to it. The value in *M* is $hgm_{i}$. The value in *V* and the control values are the first four bits of the vector $Phgm_{i}$ and the last two, respectively.

In this program, we have also included some extra affine transformations that modify the final value in *V* for heads and links. The flow and the critical points of the program are given in Figure A1.

This figure includes the information for the *H*-modifiers and the control values at each critical point. The critical points given by inputs with multiples links have *H*-modifiers for each entry, and they are not equal for all inputs. The only rule is that, at the next critical point, the *H*-modifier should be correct, and this is checked for all inputs because it depends on the values $\varphi_{g}$ and the previous *H*-modifiers.

The addition of keys has been combined with the affine transformations on each connected component, thus it is not necessary to make an explicit computation to add the key.

There are four connected components, **I**, **II**, **III** and **IV**. Each has an affine transformation that is made at the starting points of the component and restored at the ending point. The addition of the key is combined with the affine constant to get the correct result. The linear transformations and the additive constants are listed in Table A11.

**Table A11:** Affine transformations on connected components.

Connected Component	Linear Transformation	Additive Constant
**I**	$\left[\begin{array}{cccc} 0 & 1 & 0 & 1\\ 1 & 1 & 0 & 1\\ 1 & 1 & 1 & 1\\ 1 & 1 & 0 & 0 \end{array}\right]$	a
**II**	$\left[\begin{array}{cccc} 1 & 1 & 0 & 1\\ 0 & 1 & 1 & 1\\ 1 & 1 & 0 & 0\\ 0 & 0 & 1 & 1 \end{array}\right]$	8
**III**	$\left[\begin{array}{cccc} 0 & 1 & 0 & 0\\ 0 & 1 & 1 & 1\\ 1 & 1 & 0 & 0\\ 0 & 1 & 0 & 1 \end{array}\right]$	6
**IV**	$\left[\begin{array}{cccc} 0 & 1 & 0 & 0\\ 1 & 0 & 1 & 1\\ 0 & 0 & 0 & 1\\ 0 & 1 & 1 & 0 \end{array}\right]$	b

Note that it is not necessary to use an output table because, using different control values, table *B* can be reused for the output when the control value is (1,1). In this implementation, we have not used dissolving maps because the program is designed to compute combinations of heads and links and the combination of two heads it not required.

## References

[1] Chow S., Eisen P.A., Johnson H., van Oorschot P.C. (2003). A white-box DES implementation for DRM applications. Security and Privacy in Digital Rights Management, Proceedings of the ACM CCS-9 Workshop, DRM 2002, Washington, DC, USA, 18 November 2002.

[2] Jacob M., Boneh D., Felten E.W. (2003). Attacking an obfuscated cipher by injecting faults. Security and Privacy in Digital Rights Management, Proceedings of the ACM CCS-9 Workshop, DRM 2002, Washington, DC, USA, 18 November 2002.

[3] Goubin L., Masereel J.-M., Quisquater M., Adams C.M., Miri A., Wiener M.J. (2007). Cryptanalysis of white box DES implementations. Selected Areas in Cryptography, Proceedings of the 14th International Workshop, SAC 2007, Ottawa, ON, Canada, 16–17 August 2007.

[4] Chow S., Eisen P.A., Johnson H., van Oorschot P.C., Nyberg K., Heys H.M. (2002). White-box cryptography and an AES implementation. Selected Areas in Cryptography, Proceedings of the 9th Annual International Workshop, SAC 2002, St. John’s, NL, Canada, 15–16 August 2002.

[5] Billet O., Gilbert H., Ech-Chatbi C., Handschuh H., Hasan M.A. (2004). Cryptanalysis of a white box AES implementation. Selected Areas in Cryptography, Proceedings of the 11th International Workshop, SAC 2004, Waterloo, ON, Canada, 9–10 August 2004.

[6] Michiels W., Gorissen P., Hollmann H.D.L., Avanzi R.M., Keliher L., Sica F. (2008). Cryptanalysis of a generic class of white-box implementations. Selected Areas in Cryptography, Proceedings of the 15th International Workshop, SAC 2008, Sackville, NB, Canada, 14–15 August 2008.

[7] Biryukov A., Shamir A. (2010). Structural cryptanalysis of SASAS. J. Cryptol..

[8] Whibox Contest Ches 2017 Capture the Flag Challenge. https://whibox-contest.github.io/.

[9] Goubin L., Paillier P., Rivain M., Wang J. (2018). How to reveal the secrets of an obscure white-box implementation. IACR Cryptol. ePrint Arch..

[10] Biryukov A., Udovenko A., Peyrin T., Galbraith S.D. (2018). Attacks and countermeasures for white-box designs. Advances in Cryptology, Proceedings of the ASIACRYPT 2018-24th International Conference on the Theory and Application of Cryptology and Information Security, Brisbane, Australia, 2–6 December 2018.

[11] Myasnikov A.U.A., Shpilrain V. (2011). Non-Commutative Cryptography and Complexity of Group-Theoretic Problems.

[12] Nathanson M.B. (1996). Additive Number Theory. Inverse Problems and the Geometry of Sumsets.

[13] Wisbauer R. (1991). Foundations of Module and Ring Theory.

[14] Barak B., Goldreich O., Impagliazzo R., Rudich S., Sahai A., Vadhan S.P., Yang K., Kilian J. (2001). On the (im)possibility of obfuscating programs. Advances in Cryptology, Proceedings of the CRYPTO 2001, 21st Annual International Cryptology Conference, Santa Barbara, CA, USA, 19–23 August 2001.

[15] Barak B., Goldreich O., Impagliazzo R., Rudich S., Sahai A., Vadhan S.P., Yang K. (2012). On the (im)possibility of obfuscating programs. J. ACM.

[16] Castro R.R., López J., Gritzalis S. (2018). Evolution and trends in iot security. IEEE Comput..

[17] Roman R., López J., Mambo M. (2018). Mobile edge computing, fog et al.: A survey and analysis of security threats and challenges. Future Gener. Comput. Syst..

[18] Bhattacharya P.B., Jain S.K., Nagpaul S.R. (1994). Basic Abstract Algebra.

[19] Lam T.Y. (1991). A First Course in Noncommutative Rings.

[20] Aschbacher M. (1986). Finite Group Theory.

[21] Knudsen L.R., Robshaw M. (2011). The Block Cipher Companion (Information Security and Cryptography).

